# Drug resistance and vaccine target surveillance of *Plasmodium falciparum* using nanopore sequencing in Ghana

**DOI:** 10.1038/s41564-023-01516-6

**Published:** 2023-11-23

**Authors:** Sophia T. Girgis, Edem Adika, Felix E. Nenyewodey, Dodzi K. Senoo Jnr, Joyce M. Ngoi, Kukua Bandoh, Oliver Lorenz, Guus van de Steeg, Alexandria J. R. Harrott, Sebastian Nsoh, Kim Judge, Richard D. Pearson, Jacob Almagro-Garcia, Samirah Saiid, Solomon Atampah, Enock K. Amoako, Collins M. Morang’a, Victor Asoala, Elrmion S. Adjei, William Burden, William Roberts-Sengier, Eleanor Drury, Megan L. Pierce, Sónia Gonçalves, Gordon A. Awandare, Dominic P. Kwiatkowski, Lucas N. Amenga-Etego, William L. Hamilton

**Affiliations:** 1 Wellcome Sanger Institute, Wellcome Trust Genome Campus, Hinxton, UK; 2 West African Centre for Cell Biology of Infectious Pathogens (WACCBIP), College of Basic and Applied Sciences, University of Ghana, Legon, Ghana; 3 Navrongo Health Research Centre (NHRC), Ghana Health Service, Navrongo, Upper East Region, Ghana; 4 Ledzokuku Krowor Municipal Assembly (LEKMA) Hospital, Accra, Ghana; 5 Department of Medicine, University of Cambridge, Cambridge, UK; 6 Cambridge University Hospitals NHS Foundation Trust, Cambridge, UK

**Keywords:** Parasite genomics, Genomics

## Abstract

Malaria results in over 600,000 deaths annually, with the highest burden of deaths in young children living in sub-Saharan Africa. Molecular surveillance can provide important information for malaria control policies, including detection of antimalarial drug resistance. However, genome sequencing capacity in malaria-endemic countries is limited. We designed and implemented an end-to-end workflow to detect *Plasmodium falciparum* antimalarial resistance markers and diversity in the vaccine target *circumsporozoite protein* (*csp*) using nanopore sequencing in Ghana. We analysed 196 clinical samples and showed that our method is rapid, robust, accurate and straightforward to implement. Importantly, our method could be applied to dried blood spot samples, which are readily collected in endemic settings. We report that *P. falciparum* parasites in Ghana are mostly susceptible to chloroquine, with persistent sulfadoxine-pyrimethamine resistance and no evidence of artemisinin resistance. Multiple single nucleotide polymorphisms were identified in *csp*, but their significance is uncertain. Our study demonstrates the feasibility of nanopore sequencing for malaria genomic surveillance in endemic countries.

## Abstract (web summary)

Evaluation of a nanopore-sequencing-based workflow for detection of antimalarial resistance in Ghana demonstrates feasibility where molecular surveillance is needed.

## Main

Malaria is a major cause of morbidity and mortality worldwide, particularly for young children living in sub-Saharan Africa. The World Health Organization (WHO) estimates that there were 247 million malaria cases and 619,000 deaths in 2021^1^. About 76% of deaths were in children under 5 yr old and 95% were in Africa^1^. The coronavirus disease 2019 (COVID-19) pandemic disrupted malaria control services, setting back the progress made from 2000–2019^1^. The WHO has identified antimalarial drug resistance as a key threat to control and elimination efforts^2^. Artemisinin-based combination therapy (ACT) is the current front-line treatment for *Plasmodium falciparum* malaria (the most virulent species responsible for the majority of deaths). ACT is effective and well tolerated, and has been a cornerstone of progress in reducing the burden of malaria disease. Artemisinin partial resistance has been defined as delayed clearance of parasites harbouring specific mutations after treatment with an artemisinin derivative despite adequate dosing and absorption^2^. Artemisinin partial resistance, in combination with partner drug resistance, can cause treatment failure^3^. The development of ACT failure in Africa would have devastating consequences.

The capacity of parasite populations to evolve in response to interventions means that ongoing surveillance is needed to monitor for resistance. Having emerged and spread in Southeast Asia^3–12^, artemisinin partial resistance, which is caused by mutations in the gene *kelch13* (refs. ^13–15^), has now been identified in multiple east African countries including Rwanda^16–18^, Uganda^19,20^ and Eritrea^2^. Partial resistance seems to have emerged independently in Africa and Southeast Asia^16^. Resistance to sulfadoxine-pyrimethamine (SP), caused by mutations in the target genes *dhfr* and *dhps*^21–25^, threatens the efficacy of intermittent preventive therapy in pregnancy (SP-IPTp) and seasonal malaria chemoprevention (SMC) in young children (used in combination with amodiaquine, SP + AQ)^26^. SP-IPTp and SP + AQ are important public health measures to protect vulnerable populations in hyperendemic regions. Parasite genome sequencing, incorporated into surveillance programmes, has provided key information to guide National Malaria Control Programme (NMCP) decision-making. For example, the geospatial distribution and longitudinal trends of antimalarial resistance markers^27–30^ and *P. falciparum* population structure and relatedness^31–35^ were characterized using genome sequencing.

An effective vaccine is urgently needed for malaria prevention^36^. In October 2021, RTS,S/AS01 became the first malaria vaccine to be recommended by WHO for children living in areas of moderate to high *P. falciparum* transmission and is being rolled out in Ghana, Malawi and Kenya, with plans to scale up in the coming years^1,37^. The RTS,S vaccine targets circumsporozoite protein (PfCSP), which is expressed on the surface of sporozoites and is required for hepatocyte invasion^38^. RTS,S/AS01 vaccine efficacy is ~36% after four doses^39^. Another PfCSP-based vaccine, R21-M Matrix (MM), has been shown to provide up to 75% efficacy in an ongoing trial in Burkina Faso^40,41^. PfCSP is also the target for long-acting monoclonal antibodies, which show promise in prophylactic protection^42–44^. It is unclear whether diversity in the *csp* gene sequence will affect either PfCSP-based vaccines or therapeutic antibodies.

The WHO ‘Strategy to respond to antimalarial drug resistance in Africa’ (November 2022) highlights the need for strengthened surveillance capacity to increase technical and laboratory capacity and to expand coverage of data on antimalarial drug efficacy and resistance in Africa^2^. However, despite the potential of genomic sequencing for pathogen surveillance^45,46^, many endemic countries in Africa have limited capacity due to prohibitive costs, barriers to procurement, and a lack of sequencing and computing infrastructure^47^. Oxford Nanopore Technologies (ONT) is being increasingly used for rapid sequencing, diagnostics, antimicrobial susceptibility testing and epidemiological analysis in multiple pathogens, including SARS-CoV-2 (refs. ^48–51^), Zika virus^52,53^, Ebola virus^54^, chikungunya virus^55^, *Mycobacterium tuberculosis*^56–58^, and bacterial antimicrobial resistance and clinical metagenomics^59–69^. ONT devices such as the MinION are portable, relatively cheap and produce sequence data in ‘real-time’, making them well-suited to resource-limited settings including in low- and middle-income countries (LMIC). The longer sequence reads generated by ONT can provide additional advantages, such as characterizing highly polymorphic or repetitive sequences or complex structural rearrangements that are challenging to access with short reads^70,71^. ONT has lower accuracy than competitor sequencing platforms; however, the latest ONT chemistry reports single read accuracy of ≥99%^72^.

Nanopore has been used to sequence whole genomes and drug resistance genes of *P. falciparum*^73–76^. Here we demonstrate that an end-to-end nanopore sequencing workflow can be prospectively applied in an endemic setting for real-time genomic surveillance from clinical malaria samples, using the current latest ONT chemistry.

## Assay design and laboratory isolate validation

**Extended Data Fig. 1:**
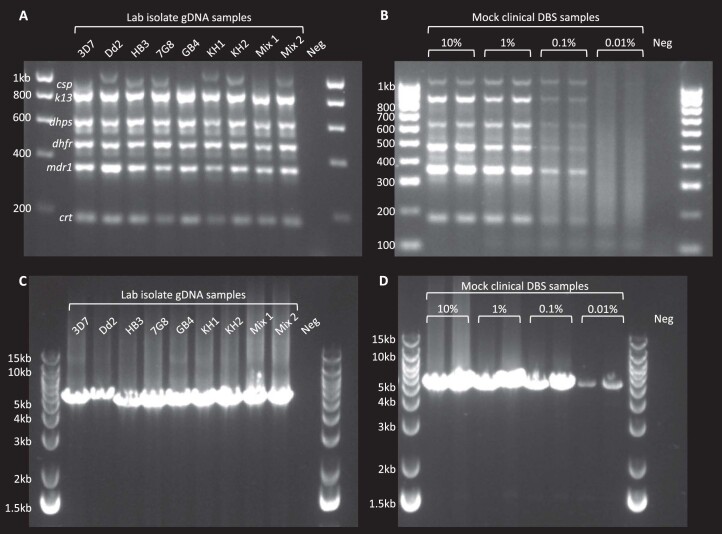
Gel electrophoresis of PCR products from laboratory clones and mock clinical samples. **a**) Multiplex drug resistance and *csp* PCR, for a selection of laboratory clones, run on 2% agarose gel. Bands are annotated based on expected sizes for each amplicon. Note variable size of *csp* due to a deletion in the N-terminal domain in 3D7 and variation in the central repeat region. Mixtures 1 and 2 contained, respectively: 3D7 + KH2 (80:20) and KH2 + 3D7 (80:20). **b**) Multiplex drug resistance and *csp* PCR, for mock clinical DBS samples, run on 2% agarose gel. Mock clinical DBS were prepared by combining in vitro cultured *P. falciparum* RBCs with human whole blood, in ratios expected to produce final parasitaemias of 10%, 1%, 0.1% and 0.01% infected RBCs, with 50 μl blotted onto filter papers to mimic clinical DBS. The proportions of human and parasite DNA per sample were assessed by quantitative PCR (Extended Data Fig. 2). Samples were extracted and assessed in duplicate. Although bands stopped being visible in the 0.01% parasitaemia samples on this gel, nanopore sequence coverage was still adequate for drug resistance genotyping. **c**) *msp1* PCR, for a selection of laboratory clones, run on 1% agarose gel. A single fragment of approximately 5Kb is expected. The same samples were used as template DNA as in gel (A). **d**) *msp1* PCR, for mock clinical DBS samples, run on 1% agarose gel. The same samples were used as template DNA as in gel (B). All template DNA was diluted to 5–10 ng/µl; 4 µl was used as input for the multiplex drug resistance and *csp* PCR, 2 µl was used as input for the *msp1* PCR, both to a final reaction volume of 50ul. 4 µl of each PCR reaction was run on the gel. DBS = Dried Blood Spots. Neg = Negative control (Nuclease Free Water used as template).

A multiplexed PCR was designed targeting six parasite loci, one amplicon within each of the antimalarial drug resistance-associated genes *chloroquine resistance transporter* (*crt*), *dihydrofolate reductase-thymidylate synthase* (*dhfr*), *dihydropteroate synthase* (*dhps*), *multidrug resistance protein 1* (*mdr1*) and *kelch13*, and the vaccine target *circumsporozoite protein* (*csp*) (Methods and Table 1). Amplicons were readily distinguished by gel electrophoresis, allowing for a cheap and straightforward check post-PCR (Extended Data Fig. 1). A separate PCR targeted the full-length sequence of the polymorphic surface antigen *merozoite surface protein 1* (*msp1*), ~5 kb in size, to further assess the potential for long nanopore reads to access complex genomic regions. A custom informatics pipeline built in Nextflow was used for real-time analysis and variant calling, referred to as ‘nano-rave’ (the Nanopore Rapid Analysis and Variant Explorer tool) (details in Methods).

**Table 1:** *P. falciparum* genes and variants targeted in amplicon assay

Gene name and ID in the 3D7 parasite clone	Key mutations targeted for genotyping	Associated antimalarial resistance or other phenotype
Chloroquine resistance transporter, *crt* (PF3D7_0709000)	K76T*	Chloroquine resistance marker
Dihydrofolate reductase, *dhfr* (PF3D7_0417200)	N51I, C59R, S108N*, I164L	Pyrimethamine resistance markers
Dihydropteroate synthase, *dhps* (PF3D7_0810800)	S436A, A437G*, K540E, A581G, A613S/T	Sulfadoxine resistance markers
Multidrug resistance protein 1, *mdr1* (PF3D7_0523000)	N86Y, N86F, Y184F	No direct inferences, but possibly associated with resistance to several antimalarials including lumefantrine
*kelch13* (PF3D7_1343700)	Different mutations in the propeller domain, for example, C580Y	Artemisinin partial resistance markers
Circumsporozoite protein*, csp* (PF3D7_0304600)	SNPs in the C-terminal region; assess full-length consensus sequence	Leading vaccine and monoclonal antibody target antigen
Merozoite surface protein 1, *msp1* (PF3D7_0930300)	Assess full-length consensus sequence	Previously explored as a vaccine candidate; potential for use as a marker of complexity of infection

A multiplex PCR targeted the drug resistance marker genes (*crt*, *dhfr*, *dhps*, *mdr1* and *kelch13*) and the vaccine and monoclonal antibody target, *csp*, in a single assay. The *msp1* PCR was performed in a separate reaction. Mutations in bold with asterisk were used as key markers of antimalarial drug susceptibility phenotyping. Details on primer sequences, amplicons and antimalarial drug susceptibility inference rules are provided in Supplementary Notes.

**Extended Data Fig. 2:**
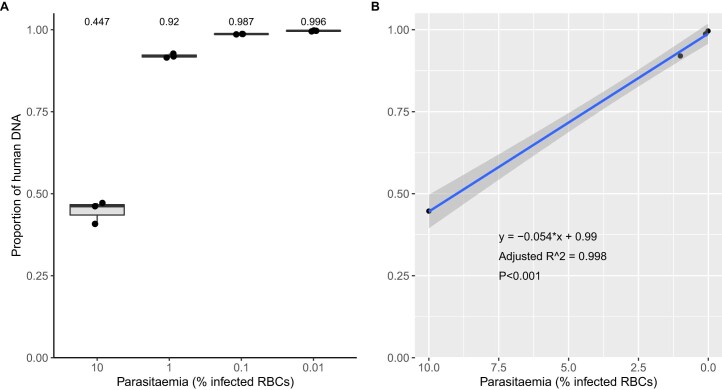
Quantitative PCR (qPCR) results for mock clinical DBS samples. RBCs with in vitro cultured *P. falciparum* parasites were added to human whole blood in proportions estimated to yield parasitaemias of 10%, 1%, 0.1% and 0.01% infected RBCs. 50ul of these samples were blotted onto filter papers to produce mock DBS. Three DBS were blotted and DNA extracted as technical replicates for each of the four parasitaemias tested (details in Methods), so total N = 12. Extracted samples underwent qPCR for human and *P. falciparum* probes, with concentrations extrapolated from the standard curve. The y-axis for both plots shows the proportion of human DNA relative to the sum of the inferred human and *P. falciparum* DNA concentrations, so values closer to 1 indicate more human DNA in proportion to parasite DNA and vice versa for values closer to zero. **a**) Boxplot of human DNA proportion for each parasitaemia (note non-linear x-axis). **b**) Linear regression model of median human DNA proportion vs parasitaemia. The grey area around the line show the standard error lines. These results confirm a strong linear relationship between the relative abundance of *P. falciparum* DNA present in the mock DBS samples and the parasitaemia estimates, with high consistency between technical replicates. Further details on qPCR conditions are described in Supplementary Notes. DBS = Dried Blood Spot; RBC = Red Blood Cell.

The workflow was applied to three sample sets: first, it was validated on laboratory parasite clones (3D7, Dd2, HB3, 7G8, GB4, KH1 and KH2) and mock clinical dried blood spot (DBS) samples, referred to collectively as ‘validation samples’ (Methods and Extended Data Fig. 2). Second, we performed prospective genomic surveillance on leucodepleted venous blood (VB) samples from clinical malaria samples collected at two sites in Ghana. Third, we retrospectively applied the workflow to a collection of DBS samples collected in northern Ghana. Nanopore sequencing was performed in multiplexed batches on an ONT MinION mk1b device using either kit 12 with R10.4 flow cells (validation and leucodepleted VB samples) or kit 14 with R10.4.1 flow cells (validation and clinical DBS samples) (Supplementary Table 1 and Methods). For the validation samples, no discrepancies were identified between the key antimalarial resistance markers genotyped in the assay and the expected genotypes for the laboratory clones tested. The lab isolate Dd2 was noted to contain both N86Y and N86F variants in *mdr1* due to having multiple copies of this gene, as previously observed (for example, ref. ^77^, discussed further in Supplementary Notes). For the two lab isolate mixtures, the consensus genotype assigned matched the expected majority clone; for example, C580Y in *kelch13*, which is associated with artemisinin partial resistance, was correctly genotyped in both the ‘pure’ KH2 isolate (known to possess that marker) and the mixture of KH2:3D7 at 80:20 ratios. However, *kelch13* was wild type with consensus genotyping for the mixture with KH2:3D7 at 20:80 ratios, as expected. Sequence reads were also mapped to the full 3D7 reference genome and manually inspected using the Integrative Genomics Viewer (IGV) tool, confirming the mixed sample at expected positions. In the mock DBS samples, drug resistance calls were concordant with the expected genotypes for the parasite clone used (Dd2), even at the lowest parasitaemias tested (predicted 0.01% infected red blood cells (RBCs)), for which bands were no longer appreciated by gel electrophoresis (Extended Data Fig. 1).

**Extended Data Fig. 3:**
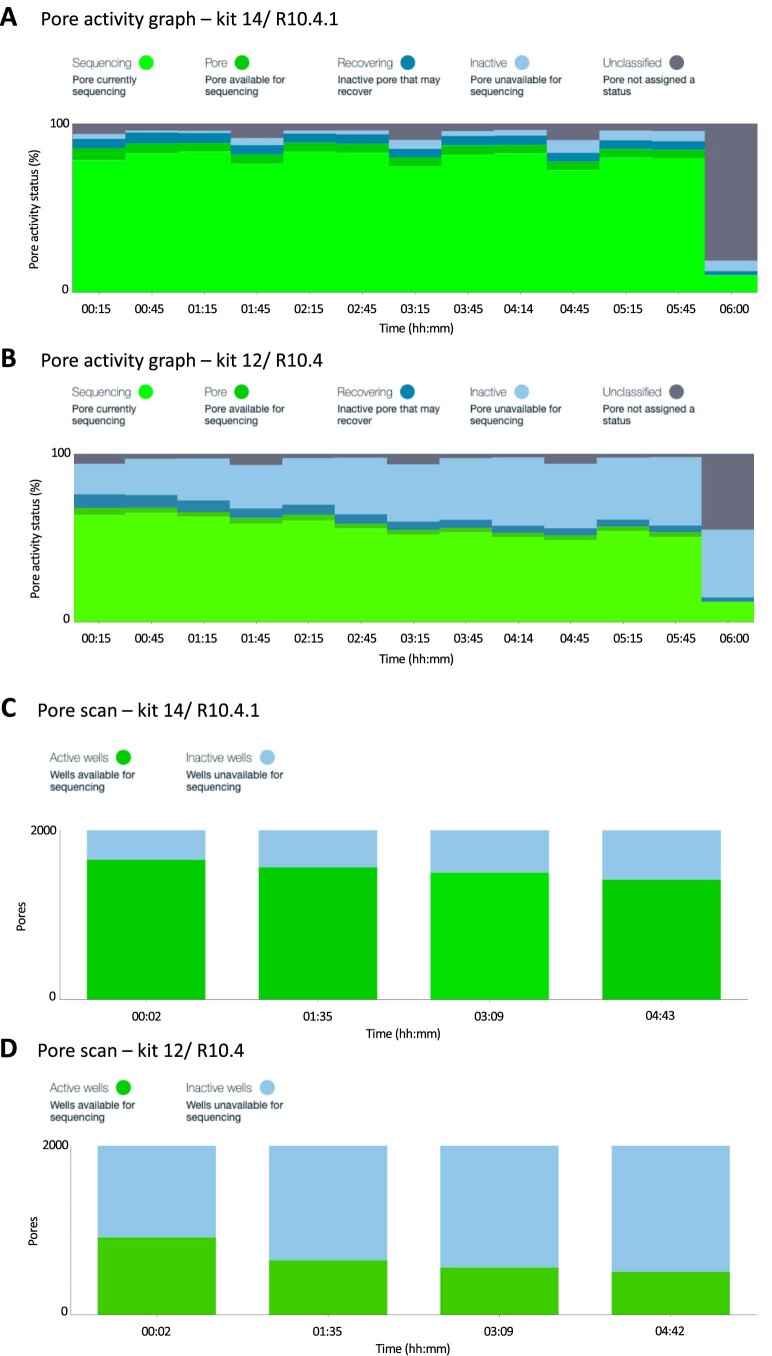
Nanopore flow cell performance comparison for kit 14/ R10.4.1 flow cells vs kit 12/ R10.4 flow cells. Nanopore flow cell performance comparison: kit 14/ R10.4.1 flow cells (**a**, **c**) vs kit 12/ R10.4 flow cells (**b**, **d**). Showing screenshots from MinKNOW depicting pore activity (top; **a**, **b**) and pore scan results (bottom; **c**, **d**). Both sequencing runs used the multiplexed validation sample set with native barcoding, and were run for 6 hours. Note the final data points for the pore activity plots is artefactual. Kit 14/ R10.4.1 is expected to produce Q20+ accuracy compared with Q20 for kit 12/ R10.4 flow cells. We have found that kit 14/ R10.4.1 flow cells produce higher and more sustained pore activity and almost no drop in pore activity after 6 hours of sequencing. Higher levels of multiplexing and/or flow cell washes and re-use would therefore be an option to reduce costs per sample.

The validation samples were sequenced using both kit 12/R10.4 flow cells and kit 14/R10.4.1 flow cells. Relative to R10.4 flow cells, we observed improved flow cell performance over the course of sequencing using the R10.4.1 flow cells (expected Q20+ accuracy) (Extended Data Fig. 3), with increased total data generated from a 6 h run (52 GB vs 39 GB), estimated bases (2.76 Gb vs 1.86 Gb), reads generated (3.47 M vs 2 M) and base-called pass bases (real-time super-accurate ‘guppy’ base calling; 2.57 M vs 1.61 M) (Supplementary Table 1). These trends were consistent for multiple R10.4.1 flow cells.

## Clinical sample collection and study population

**Fig. 1:**
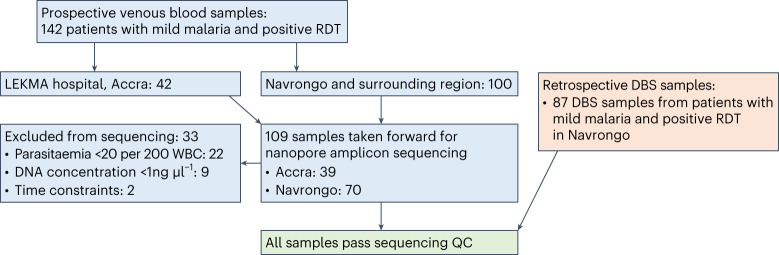
Study flow diagram. For the prospective collection of leucodepleted VB samples, 142 patients with malaria symptoms and positive RDT were recruited to the study from LEKMA Hospital in Accra or three clinics in and around Navrongo in the Upper East Region. Samples were excluded if parasitaemia was <20 per 200 WBC, DNA concentration post-extraction was <1 ng µl^−1^, or due to time constraints (20 parasites per 200 WBC corresponds to ~1,000 parasites per µl blood or 0.03% infected RBCs). A total of 109 samples were included in the study and taken forward for nanopore sequencing. The workflow was also tested on a retrospective set of 87 DBS samples. The DBS were collected in 2018 from in and around Navrongo, and extracted DNA had been stored frozen. DBS samples were selected that had passed MalariaGEN quality control filtering for Illumina whole-genome sequencing, with parasitaemia >1 parasite per 200 WBC and post-extraction DNA concentration of >1 ng ul^−1^. All samples, for all amplicons, produced >50× sequence coverage and were therefore included in downstream analyses.

**Extended Data Fig. 4:**
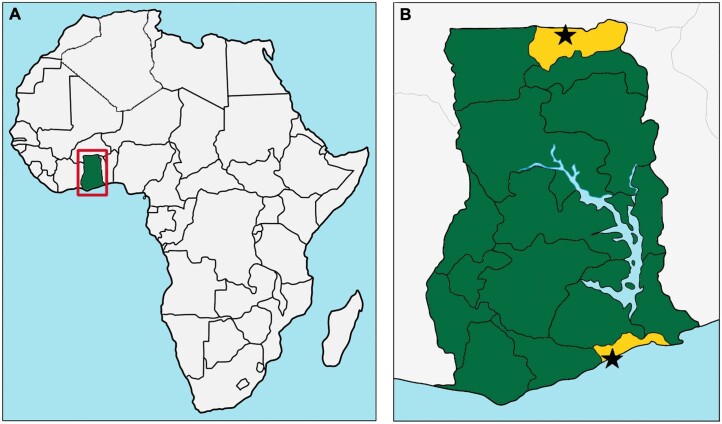
Map showing location of Ghana in West Africa and the two field sites within Ghana where the study was based. Map showing location of Ghana in West Africa (**a**) and the two field sites within Ghana where the study was based (**b**), indicated by black stars: LEKMA Hospital in Accra, near the coast, and Navrongo in the Upper East Region.

Prospective clinical sample collection took place in two locations in Ghana, one urban (LEKMA Hospital, Accra, on the coast) with perennial malaria transmission and one rural (three sites in and around Navrongo, in the Upper East Region), where malaria transmission is highly seasonal (Extended Data Fig. 4). Samples were collected from August to September 2022 during the rainy, high-transmission season. Patients (142) with a positive *P. falciparum* rapid diagnostic test (RDT) were recruited to the study; 42 from LEKMA Hospital and 100 from Navrongo (Fig. 1). Samples were typically 0.5–2 ml venous blood that underwent leucodepletion by centrifugation and Buffy coat removal (Methods). Samples from 33 patients were excluded from nanopore sequencing due to low parasitaemia (<20 parasites per 200 white blood cells, WBC), poor DNA yield post-extraction (<1 ng µl^−1^) or time constraints. This yielded a final sample set of 109 samples, 70 from Navrongo and 39 from Accra, which were taken forward for nanopore sequencing and analysis.

**Extended Data Fig. 5:**
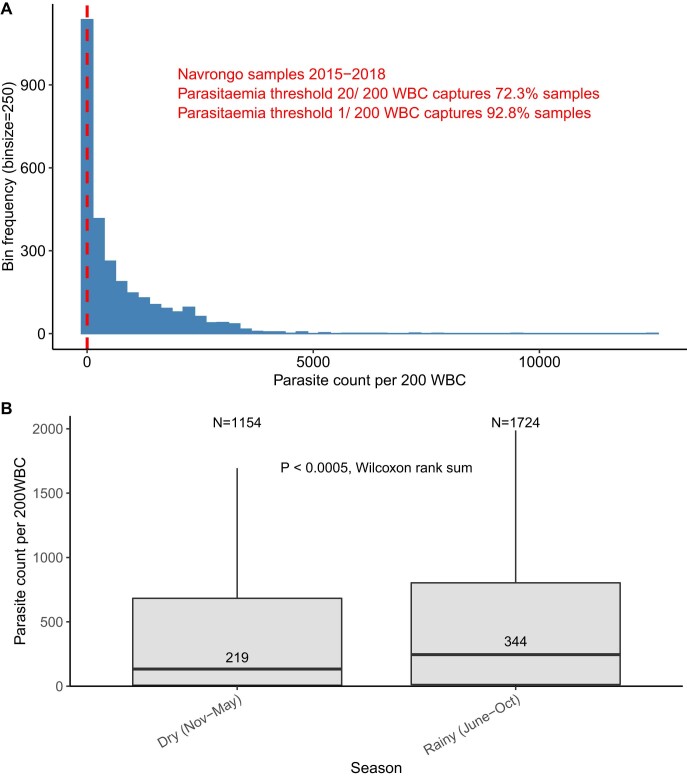
Parasitaemia distribution for mild malaria cases sampled in the Upper East Region of northern Ghana, 2015–2018. Parasitaemia distribution for mild malaria cases sampled in the Upper East Region of northern Ghana, 2015–2018 (data gathered by Dr Lucas Amenga-Etego in previous studies). Cases were identified as having symptoms compatible with malaria and a positive rapid diagnostic test (RDT). (**a**) Histogram of parasitaemias for all samples (n = 2,878), indicating the minimum threshold used for the leucodepleted venous blood samples in this study of 20 parasites per 200 white blood cells (WBC) with dashed red line. Using this cut-off, 72.3% of all samples would have been included. For the dried blood spot (DBS) samples analysed in this study, we applied a cut-off of requiring microscopy positivity that is at least 1 parasite per 200 WBC. This would capture 92.8% of all samples from the Navrongo cohort shown in this figure, with similar values for both Dry and Rainy seasons. (**b**) Box plot of parasitaemias separated into samples collected during the Dry and Rainy seasons in Navrongo, defined roughly as November – May (n = 1154) and June – October (n = 1724), respectively. Transmission intensity is substantially higher during the Rainy season compared with the Dry season; consistent with this, a tendency for higher parasitaemias during the Rainy season is observed (median parasite count of 219 vs 344 in Dry vs Rainy seasons, respectively (*P* < 0.0005, two-sided Wilcoxon rank sum test)). The cut-off of 20 parasites per 200 WBC would capture 69% and 74.6% of samples from the Dry and Rainy seasons, respectively. WBC = White Blood Cells.

For the 109 samples taken forward, median patient age was 12 yr old (interquartile range (IQR) 5–22 yr). There were 54 females and 51 males (4 unrecorded). Median parasite count was 864 (IQR: 243–1,582) per 200 WBC, corresponding to ~43,000 parasites per µl blood (IQR: 12,000–79,000) or 1.4% infected RBCs (IQR: 0.4%–2.6%). The lowest parasitaemia included was 21 parasites per 200 WBC, or ~1,000 parasites per µl blood (~0.03% infected RBCs). For clinical samples collected in another study from 2015–2018 from mild malaria cases in Navrongo^22^, a parasitaemia cut-off of 20 parasites per 200 WBC would have included 72.3% of all samples (Extended Data Fig. 5).

## Multiplexed nanopore sequencing of venous blood samples

**Extended Data Fig. 6:**
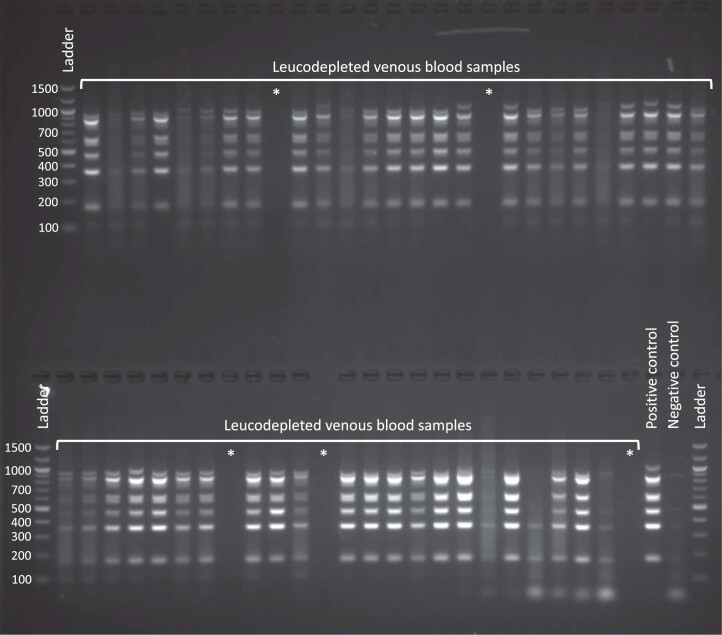
Gel electrophoresis image of leucodepleted venous blood samples collected from patients with malaria at LEKMA Hospital, Accra, following multiplex drug resistance and *csp* PCR. Top: ladder in lane 1; samples in lanes 2–28 except for empty wells in lanes 10 and 19. Bottom: ladder in lanes 1 and 29; samples in lanes 2–25 except for empty wells in lanes 9, 13 and 26; positive control (pure *P. falciparum* gDNA) in lane 27; negative control (nuclease free water) in lane 28. Asterisks indicate empty lanes. Fragment size distribution follows the expected pattern, and is consistent with the laboratory and mock clinical isolates shown in Extended Data Fig. 1.

All of the 109 venous blood samples included were used for the multiplex drug resistance and *csp* PCR amplification assay, with encouraging gel electrophoresis results (Extended Data Fig. 6). Using kit 12/R10.4 flow cells, 6–8 h of sequencing on the MinION mk1b in multiplexed batches of ~24 samples per flow cell produced a median of 34 GB data, 1.62 Gb bases, 1.73 M reads and 1.26 Gb pass bases called per run (Supplementary Table 1). Real-time base calling was performed using the graphics processing unit (GPU) of a commercial gaming laptop and the resulting fastq files were used directly for downstream analysis.

**Fig. 2:**
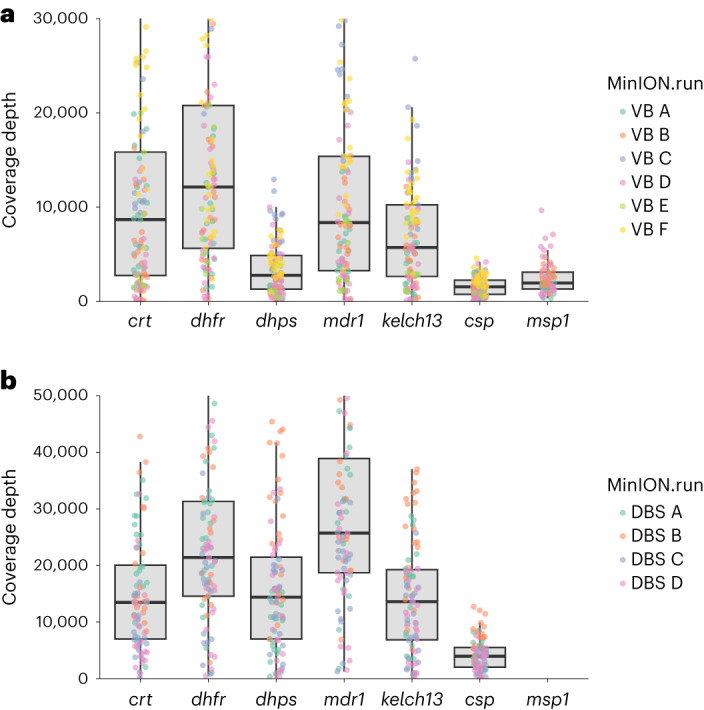
Coverage profile of amplicon targets. **a**, Leucodepleted VB samples, sequenced with kit 12 chemistry/R10.4 flow cells (*N* = 109 samples). The *y* axis shows median number of reads covering each amplicon target per sample for each of the MinION runs for clinical samples. Median coverages for the *crt*, *dhfr*, *dhps*, *mdr1*, *kelch13*, *csp* and *msp1* amplicons were 8,682, 12,141, 2,772, 8,369, 5,727, 1,552 and 1,957, respectively. **b**, DBS samples (*N* = 87) sequenced with kit 14 chemistry/R10.4.1 flow cells. In both figures, positive controls and sample duplicates were excluded. Coverage data were derived from BEDTools produced in the ‘nano-rave’ pipeline. Note that the *msp1* PCR was only included in the leucodepleted VB samples from Navrongo (70/109), so coverage is only shown for these samples. Coverage was higher for the DBS samples; however, this may reflect using the newer ONT chemistry and flow cells.

The ‘nano-rave’ pipeline can be run directly from the demultiplexed, base-called fastq files and folder organization created in real-time during each ONT flow cell sequencing run, allowing rapid analysis. Median coverage across the amplicon targets was greater than 1,000× per sample for all amplicons (range: 1,552× median coverage for *csp* to 12,141× for *dhfr*) (Fig. 2), suggesting substantial scope for increased multiplexing to reduce costs. No amplicons from any sample in the 6–8 h runs had <50× coverage, and therefore all samples were included in downstream genetic analyses; this suggested that lower parasitaemias and non-leucodepleted lower-volume blood samples (such as DBS) could be used as sample input, which we subsequently confirmed (discussed below).

To streamline the workflow and reduce informatic requirements, we aimed to genotype single nucleotide polymorphisms (SNPs) using majority consensus calls, that is, for genotypes from samples with mixed infections (more than one parasite clone present in the sample) to be based on the genotype of the most abundant clone. Several variant calling tools are available through the nano-rave pipeline: ‘medaka variant’, ‘medaka haploid’^78^ and ‘freebayes’^79^ (see further information in Supplementary Notes). ‘Medaka haploid’ was the fastest of these variant callers and felt to be the best suited for producing majority genotype calls from a haploid genome with nanopore reads and potential for mixed infections. For 14 venous blood samples, the workflow from PCR through to sequencing and variant calling was repeated to assess for assay consistency. No discrepancies were observed between the repeated samples using ‘medaka haploid’ variant calling from ‘guppy’ super-accuracy or high-accuracy base-called reads, enabling these genotypes to be used for downstream analysis.

## Multiplexed nanopore sequencing from DBS samples

**Extended Data Fig. 7:**
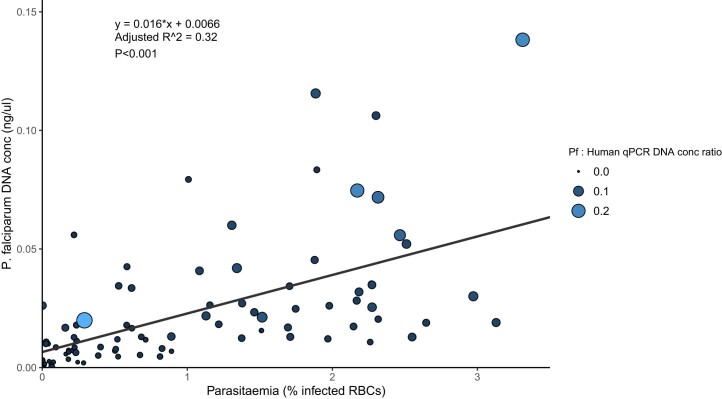
*P. falciparum* DNA concentration measured by qPCR versus parasitaemia for dried blood spot samples collected from Navrongo. *P. falciparum* DNA concentration measured by qPCR versus parasitaemia (percentage of infected RBCs) measured by microscopy, for the 87 dried blood spot (DBS) samples collected from Navrongo and sequenced with ONT kit 14 chemistry/ R10.4.1 flow cells in this study. Point size and colour are proportional to the ratio of *P. falciparum* (Pf) to human DNA concentration inferred by qPCR. Linear regression shows a significant positive relationship between parasitaemia (%) and sample *P. falciparum* DNA concentration, though with greater variability than for the mock DBS samples (Extended Data Fig. 2). qPCR methods are described in Supplementary Notes.

DBS samples are less invasive and more easily collected than VB samples in resource-limited settings. The capacity to sequence directly from DBS samples would substantially extend the potential applicability of our workflow for malaria genomic surveillance in endemic countries. We tested the workflow using the multiplex drug resistance marker and *csp* PCR (with minor modifications to the PCR conditions, described in Methods) retrospectively on a set of 87 microscopy-positive DBS samples collected from Navrongo in 2018. These samples were collected as part of ongoing surveillance work in the region and already known to pass MalariaGEN quality control filters with Illumina whole-genome sequencing (described in ref. ^80^). Median parasitaemia for the samples was 713 per 200 WBC (IQR 219–1,882); the lowest-parasitaemia sample had 2 parasites per 200 WBC (~100 parasites per μl blood), that is, close to the limit of microscopy positivity. As expected, lower-parasitaemia samples had less *P. falciparum* and more human genomic DNA (gDNA) detected by quantitative PCR (Extended Data Fig. 7). Kit 14/R10.4.1 flow cells were used in multiplexed batches of 24 samples per flow cell, run for 6–8 h on a MinION mk1b device, with real-time super-accurate ‘guppy’ base calling and genotyping using medaka haploid in the nano-rave pipeline. Each flow cell run included a positive and negative control and a single sample was sequenced twice to compare between-run consistency.

**Extended Data Fig. 8:**
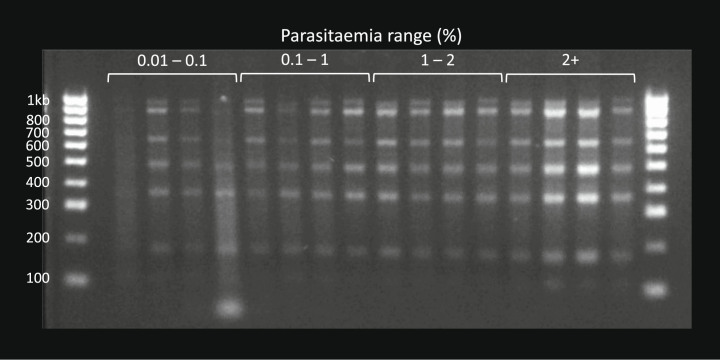
Gel electrophoresis image of dried blood spot samples collected from Navrongo, following multiplex drug resistance and *csp* PCR. Gel electrophoresis image of dried blood spot (DBS) samples collected from Navrongo, following multiplex drug resistance and csp PCR. Ladder in lane 1; samples organised left to right by increasing parasitaemias. Bands are visible at the expected sizes even for the lowest parasitaemias. Fragment size distribution follows the expected pattern, and is consistent with the laboratory and mock clinical isolates shown in Extended Data Fig. 1 and the leucodepleted venous blood samples shown in Extended Data Fig. 6.

**Extended Data Fig. 9:**
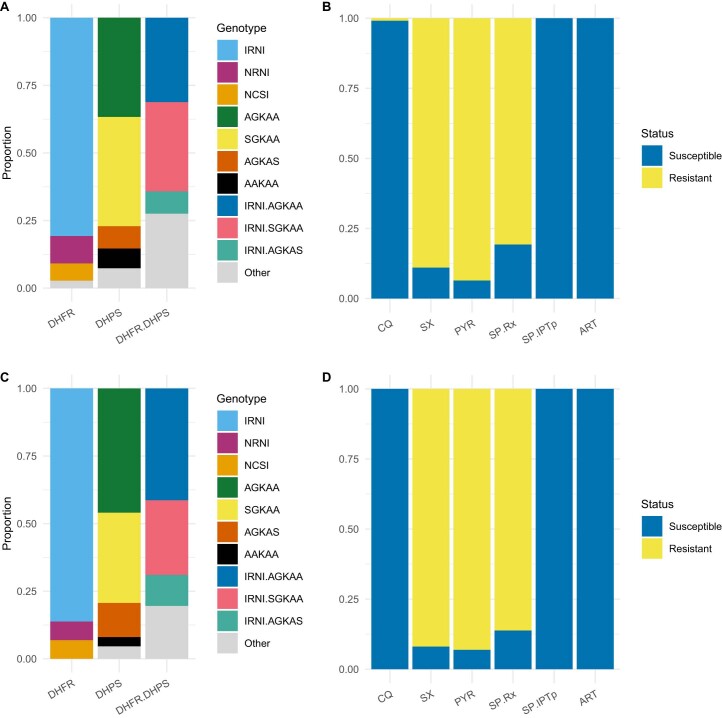
DHFR and DHPS haplotypes and inferred antimalarial susceptibility profile for leucodepleted venous blood samples and dried blood samples. DHFR and DHPS haplotypes (**a**) and inferred antimalarial susceptibility profile (**b**) for the 109 leucodepleted venous blood samples; and DHFR and DHPS haplotypes (**c**) and inferred antimalarial susceptibility profile (**d**) for the 87 dried blood spot samples. DHFR = Dihydrofolate reductase; DHPS = Dihydropteroate synthase; CQ = Chloroquine; SX = Sulfadoxine; PYR = Pyrimethamine; SP.Rx = Combination Sulfadoxine-Pyrimethamine (SP) as treatment for symptomatic malaria; SP.IPTp = Combination SP for intermittent preventive therapy in pregnancy; ART = Artemisinin. DHFR haplotypes refer to amino acid positions 51, 59, 108 and 164 (wild-type = NCSI). DHPS haplotypes refer to amino acid positions 436, 437, 540, 581 and 613 (fully susceptible = SAKAA). Inference rules for (**b**) and (**d**) are shown in Supplementary Notes. Note that for artemisinin, ‘resistance’ refers to artemisinin partial resistance (defined in main text). The leucodepleted venous blood and dried blood spot sample data are combined in Fig. 3 of main text.

Consistent with the mock DBS samples, bands were visible post-PCR by gel electrophoresis down to very low parasitaemias (Extended Data Fig. 8). Amplicon coverage was high (Fig. 2b); all amplicons had at least 50× coverage, including the samples with parasitaemias of <20 parasites per 200 WBC and so were included in downstream analysis. Antimalarial resistance marker frequencies were consistent between the VB and DBS samples (Table 2 and Extended Data Fig. 9). The workflow was repeated twice for a single DBS sample, from PCR to sequencing, and again no discrepancies between repeats were identified. These data suggest that ONT can be used for amplicon sequencing of *P. falciparum* directly from DBS samples even at very low (but still microscopy-positive) parasitaemias, without requiring a selective whole-genome amplification step.

**Table 2:** Antimalarial drug resistance genetic marker frequencies for the combined 196 samples (109 leucodepleted VB and 87 DBS samples)

Gene	SNP	Accra VB, n (%), Total *N* = 39	Navrongo VB, n (%), Total *N* = 70	Navrongo DBS, n (%), Total *N* = 87	All, n (%), Total *N* = 196
*crt*	K76T	1 (2.6%)	0	0	1 (0.5%)
*dhfr*	N51I	36 (92.3%)	54 (77.1%)	75 (86.2%)	165 (84.2%)
*dhfr*	C59R	37 (94.9%)	62 (88.6%)	81 (93.1%)	180 (91.8%)
*dhfr*	S108N	38 (97.4%)	64 (91.4%)	81 (93.1%)	183 (93.4%)
*dhfr*	S108T	0	0	0	0
*dhfr*	I164L	0	0	0	0
*dhfr*	I164M	0	0	0	0
*dhps*	S436A	19 (48.7%)	42 (60.0%)	54 (62.1%)	115 (58.7%)
*dhps*	S436F	1 (2.6%)	0	2 (2.3%)	3 (1.5%)
*dhps*	A437G	36 (92.3%)	61 (87.1%)	80 (92%)	177 (90.3%)
*dhps*	K540E	0	0	0	0
*dhps*	K540N	0	0	0	0
*dhps*	A581G	2 (5.1%)	2 (2.9%)	0	4 (2.0%)
*dhps*	A613S	6 (15.4%)	8 (11.4%)	13 (14.9%)	27 (13.8%)
*dhps*	A613T	0	0	0	0
*mdr1*	N86Y	0	2 (2.9%)	1 (1.2%)	3 (1.5%)
*mdr1*	Y184F	27 (69.2%)	49 (70.0%)	63 (72.4%)	139 (70.9%)
*kelch13*	-	0	0	0	0

The table shows sample counts for the non-reference allele for each SNP and non-reference allele frequency in brackets. Denominators are 39 VB samples from Accra, 70 venous blood samples from Navrongo, 87 DBS samples from Navrongo and 196 samples in total. For *kelch13*, all mutations were investigated and although several SNPs were identified, these were not known to be associated with artemisinin partial resistance (details in main text).

## Drug-resistance marker frequencies

**Fig. 3:**
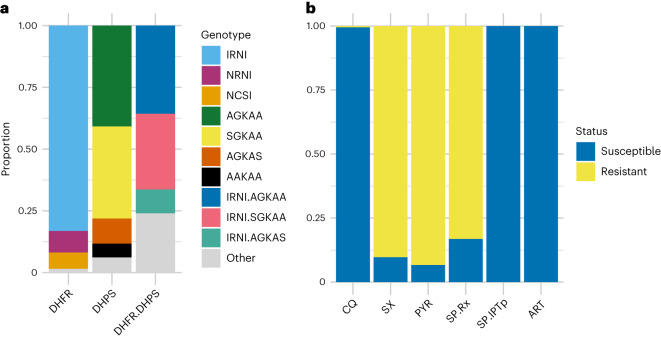
DHFR and DHPS haplotypes and inferred antimalarial resistance frequencies. **a**,**b**, DHFR and DHPS haplotypes (**a**) and inferred antimalarial resistance frequencies (**b**) for the combined 196 samples (109 leucodepleted VB and 87 DBS samples). CQ, chloroquine; SX, sulfadoxine; PYR, pyrimethamine; SP.Rx, combination sulfadoxine-pyrimethamine (SP) as treatment for symptomatic malaria; SP.IPTp, combination SP for intermittent preventive therapy in pregnancy; ART, artemisinin. DHFR haplotypes refer to amino acid positions 51, 59, 108 and 164 (wild type, NCSI). DHPS haplotypes refer to amino acid positions 436, 437, 540, 581 and 613 (fully susceptible, SAKAA). Inference rules for **b** are shown in Supplementary Notes. Note that for artemisinin, ‘resistance’ refers to artemisinin partial resistance (defined in main text). The leucodepleted VB and DBS sample data are displayed separately in Extended Data Fig. 9.

Antimalarial susceptibility was inferred from SNP genotypes using previously described inference rules^11^ (Table 2, Fig. 3 and Supplementary Notes). Combining the prospective VB (*n* = 109) and retrospective DBS samples (*n* = 87) to yield a combined analysis set of 196 samples, we found that the vast majority (>99%) of samples were chloroquine susceptible, with only a single sample carrying the resistant *crt*-76T allele. There were high frequencies of resistant alleles to pyrimethamine (93.4% *dhfr*-108N) and sulfadoxine (90.3% *dhps*-437G). The majority genotype combination (for simplicity referred to as haplotype; see caveats in Discussion) in *dhfr* was **IRN**I (83.2%)—the ‘triple mutant’—referring to amino acid positions 51, 59, 108 and 164 (wild type, NCSI; mutant positions in bold and underlined). About 8.7% of samples were N**RN**I—the ‘double mutant’, or others (8.2%). No samples carried the highly resistant *dhfr*-164L allele. The two main *dhps* haplotypes identified were **AG**KAA (40.8%) and S**G**KAA (37.2%), that is (**A**/S)**G**KAA accounted for 78.1% of samples; this refers to *dhps* amino acid positions 436, 437, 540, 581 and 613 (fully susceptible, SAKAA; note that the 3D7 reference clone carries the resistant allele S**G**KAA). The most common *dhfr* and *dhps* haplotype combinations were *dhfr*-**IRN**I + *dhps*-**AG**KAA (35.7%) or *dhfr*-**IRN**I + *dhps*-S**G**KAA (30.6%). The haplotype combinations specifically associated with declining efficacy of SP-IPTp were not observed (defined as *dhfr*-51I, *dhfr*-59R, *dhfr*-108N + *dhps*-437G, *dhps*-540E + either *dhfr*-164L or *dhps*-581G or *dhps*-613S/T). However, all of these mutations except for *dhps*-540E were observed in this sample set. The prevalence of *dhps*-581G and *dhps*-613S mutations were 2.0% and 13.8%, respectively, and the *dhfr*-**IRN**I + *dhps*-**AG**KA**S** combination was present in 9.7% of samples. In *mdr1*, the frequency of the 86Y mutation was very low (3/196), while the 184F allele frequency was 70.9% (139/196).

No mutations in *kelch13* were identified that have previously been associated with artemisinin resistance. Nine *kelch13* mutations were identified, five synonymous changes (two samples with C469C, and samples carrying T478T, A627A and S649S) and four non-synonymous mutations: A578S, Q613H, N629Y and V637I, all of which have previously been reported in Africa^81,82^ and are not considered to be associated with artemisinin resistance (Supplementary Notes).

## Antigens and vaccine targets

**Fig. 4:**
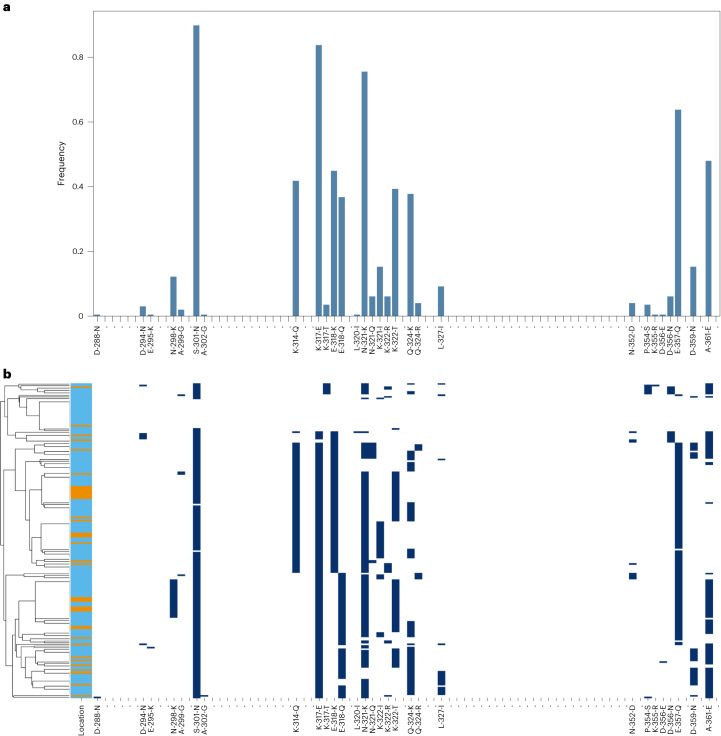
SNP frequencies in the *csp* CTR. **a**, Frequencies of SNPs along the CTR of *csp* identified from the nanopore data (combining both the VB (*N* = 109) and DBS (*N* = 87) samples, for a total of 196 analysed samples), relative to the 3D7 reference sequence. Amino acid positions 288 to 362 (in the 3D7 reference) are displayed left to right, ordered from N terminal to C terminal. Only variants with at least one sample carrying the non-reference allele are named on the *x* axis. **b**, Amino acid changes for the *csp* CTR for each sample in the study (*N* = 196, rows), where dark blue indicates the non-reference allele for that sample at that position. Samples in **b** are grouped by haplotype similarity as represented by the dendrogram (left), with the colour bar indicating whether the sampling location was Accra (orange) or Navrongo (sky blue). Note that the *csp* CTR spans from amino acids 273 to 397 in the 3D7 reference; however, no variants were identified in this cohort outside of the amino acids 288 to 362 displayed in the figure.

**Extended Data Fig. 10:**
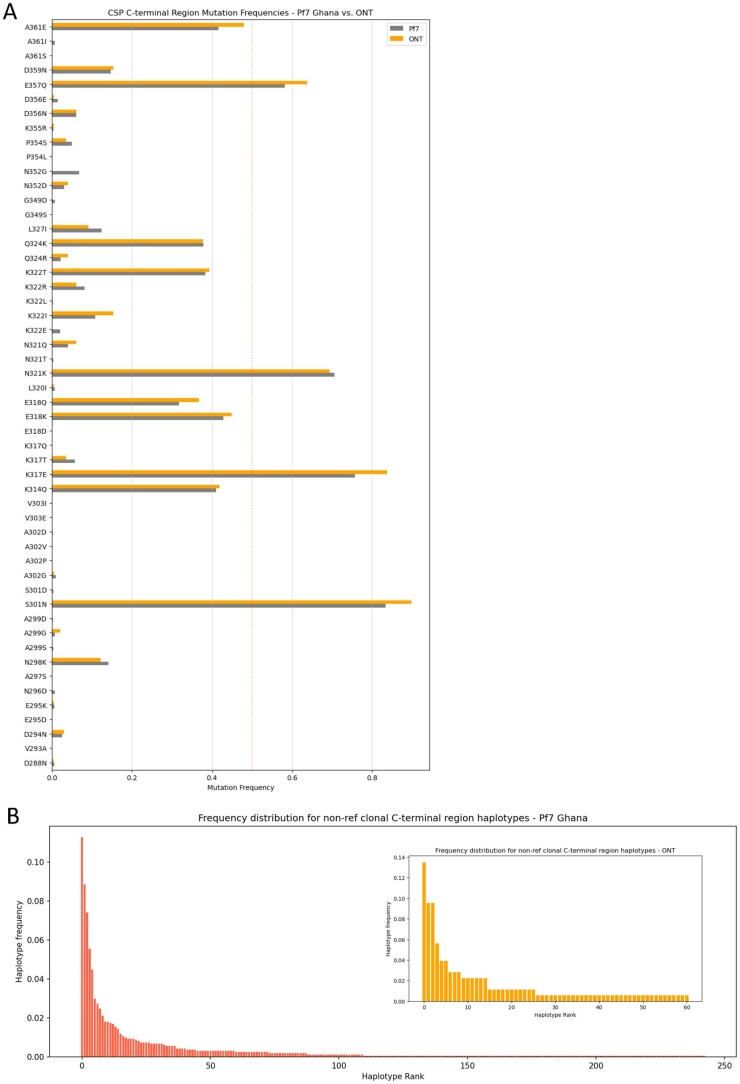
Comparison of nanopore amplicon (this study) vs. whole genome Illumina sequence data (from the MalariaGEN Pf7 data resource) for describing *csp* diversity. Comparison of amplicon ONT (this study) vs. whole genome Illumina sequence data (from the MalariaGEN Pf7 data resource) for describing *csp* diversity. **a**. Allele frequency estimates in Ghana for mutations in the C-terminal region (CTR) of the *csp* gene. Frequency estimates for the ONT data generated in this study (ONT, n = 196) are very close to the estimates produced by the Ghanian samples of the MalariaGEN Pf7 dataset (WGS, n = 1746). This analysis uses all C-terminal mutations observed in both datasets (selecting only samples from Ghana in Pf7) within clonal haplotypes (that is, heterozygous mutation calls in Pf7 samples were discarded for frequency estimation). We also discarded Pf7 samples with missing data for the C-terminal haplotype. For several SNPs, the ONT samples produced a higher non-reference allele frequency (NRAF) estimate than in Pf7. However, we confirmed with Fisher’s exact tests (2-sided) that the frequency differences could be explained by the variance introduced by the smaller ONT sample size. All 17 SNPs with NRAF > 5% in the ONT data were below the *p*-value threshold, set using Bonferroni correction for multiple comparisons (0.05/17 = 0.0029). The lowest *p*-value was for K317E (*p* = 0.0075), and in this context the allele frequency change (0.76 in Pf7 to 0.84 in the ONT data) is unlikely to be meaningful. All other SNPs had *p*-values > 0.01. **b**. Non-reference haplotype frequency distributions for the *csp* CTR in samples from Ghana. We compare the ONT samples from this study (inset; ONT, n = 178) with Ghanian samples in the Pf7 dataset (WGS, n = 1604), after removing missing, heterozygous and reference haplotypes (that is, haplotypes without any allele difference from the reference). Both distributions have a very similar shape, with a small set of high-frequency haplotypes that quickly decay into a long tail of minor ones. In addition, the first and third top-ranking haplotypes in both datasets are identical. This figure indicates that not only that CTR mutations have very similar frequencies in both datasets, but that haplotype distribution and composition are also alike.

We investigated SNP diversity in the C-terminal region (CTR) of *csp*, which is included in both the RTS,S/AS01 and R21-MM vaccines. Multiple SNP differences from the vaccine reference sequence were identified at high frequencies (>50% samples), resulting in amino acid changes such as S301N, K317E, E318(K/Q), N321K and E357Q (Fig. 4 and Supplementary Table 2). The 301N mutation was present in 90% of samples. These SNP frequencies agreed very closely with whole-genome sequence data using Illumina for *P. falciparum* in Ghana from the MalariaGEN Pf7 data resource^80^ (Extended Data Fig. 10). There was no evidence of population structure between the *csp* CTR haplotypes present in Accra and Navrongo (Fig. 4b). Overall, just 18 (9.2%) samples did not have any SNP mutations identified in the *csp* CTR relative to the vaccine sequence. Parasites carrying an exact match to the RTS,S/AS01 or R21-MM *csp* haplotype were therefore a small minority of the parasite population in Ghana. However, our study did not assess whether the variants identified have any effect on vaccine efficacy.

Lastly, we assessed nanopore sequencing for production of accurate consensus full-length amplicon sequences of *csp* and *msp1* in the validation samples. The Amplicon_sorter tool^83^ was used to produce consensus sequences with a similarity threshold of 96% (details in Methods). Consensus sequences produced from PCR products in the expected ~5 Kb size range of the *msp1* amplicon (covering almost the entire *msp1* gene) had 100% base-perfect mapping back to the reference sequences for all of the laboratory clones tested. For *csp*, base-perfect consensus sequences were generated for the clones 3D7, Dd2, HB3, GB4 and KH2. Discrepancies were observed in the number of repeats in the central repeat region for two clones: in 7G8 there was a 12 bp deletion relative to the reference sequence (ATGCAAACCCAA). In KH1 there was a 24 bp insertion relative to the reference (GCAAACCCAAATGCAAACCCAAAT). It is possible that the reference sequences for these isolates were incorrect, or that the clones used for this experiment had altered during in vitro division relative to those used to produce the reference sequences.

## Discussion

We implemented an end-to-end nanopore sequencing workflow for *P. falciparum* using standard molecular biology equipment, a handheld MinION device and a commercially available laptop computer on clinical samples in Ghana. A multiplexed PCR approach targeting key antimalarial drug resistance markers and almost full-length *csp* produced actionable data rapidly, accurately and cheaply, with a turnaround time of ~2 d from sampling to analysis outputs. Our report demonstrates the feasibility of using nanopore sequencing in endemic countries for targeted malaria molecular surveillance.

Our workflow was effective using both venous blood and dried blood spot samples, down to the lowest microscopy-positive parasitaemias. Parasitaemia of >1 parasite per 200 WBC (~100 parasites per μl blood) would be expected to capture the majority (>90%) of symptomatic malaria cases in Navrongo (Extended Data Fig. 5). Given the high depth of sequence coverage for most samples, increased sample multiplexing per MinION flow cell would very likely be successful, increasing throughput and reducing costs. After relatively modest up-front hardware expenses, we estimate running costs of around US$35 per sample with multiplexed batches of 96 samples.

Chloroquine resistance was highly prevalent (>80%) in Ghana in the early 2000s^84^. Our data indicate a trend towards increased chloroquine susceptibility. This most probably reflects shifts in national treatment policy, as chloroquine was phased out due to resistance and ACT became the front-line antimalarial treatment in Ghana in 2005. Increasing chloroquine susceptibility has also been observed in Malawi^85^. In West Africa, the pattern is variable, with contrasting chloroquine resistance rates observed in nearby countries^80^. The high prevalence of *dhfr*-**IRN**I triple mutant (83%) parasites is broadly consistent with previous results from northern Ghana^22^, in which the *dhfr*-**IRN**I triple mutant frequency was 67.9% in 2018. We also observed a high prevalence (78%) of *dhps*-(S/**A**)**G**KAA parasites. Although SP is no longer used for malaria treatment in Ghana, SP + AQ was introduced in 2016 for SMC targeting young children aged 3–59 months during the high-transmission season in northern Ghana, and SP is also used as a prophylaxis in pregnancy (IPTp). Thus, there is continued parasite exposure to SP, which may be contributing to sustained and/or increasing resistant alleles in *dhfr* and *dhps*. There was no evidence of the high-level SP resistance marker *dhps*-540E that has been associated with reducing IPTp efficacy^25^. However, several other concerning mutations such as *dhps*-581G and *dhps*-613S were identified. No *kelch13* mutations associated with artemisinin partial resistance were identified. Ongoing molecular surveillance of markers for SP, artemisinin and partner drug resistance remains critical in Africa.

The *csp* CTR harbours multiple SNPs relative to the reference sequence used in the RTS,S vaccine^86–92^, and the more polymorphic regions correspond to T-cell epitopes^93^. The relationship between genetic diversity in *csp* and the efficacy of *csp*-based vaccines and monoclonal antibody therapies is incompletely understood, with conflicting findings for RTS,S (for example, refs. ^90^ and ^94,95^). While our study does not address this question, it demonstrates that nanopore is an effective method for genotyping SNPs in the *csp* CTR as part of a multiplex surveillance panel. The SNP frequencies identified using ONT are very consistent with whole-genome sequence data generated using Illumina technology in Ghana^80^. Future work can assess whether specific *csp* genetic variants have an effect on vaccine efficacy.

Sequencing workflows that can be implemented in endemic settings are essential to (1) drive the decentralization of genomics, (2) support its integration into clinical and public health applications and (3) push for a more equitable distribution of global genomics capacity. Amplicon sequencing can be a pragmatic approach to malaria molecular surveillance^11,12,96–108^. A key factor to ensure that genomics can be deployed in endemic countries is to have stable supply chains for the procurement of laboratory consumables and sequencing hardware, and post-purchase technical support. Despite recent efforts by the Africa CDC Pathogen Genomics Initiative (PGI) to ease supply chain barriers, improved delivery of sequencing reagents across Africa and ensuring equitable access remain major challenges.

Our study has several limitations. The nano-rave informatics workflow was designed to be streamlined and rapid, and does not attempt to deconvolute mixed infections, making it unreliable to infer haplotypes (that is, genotypes shared within each clone). Copy number variation (CNV) was not assessed in this assay, such as amplifications in the drug resistance markers *mdr1* or *plasmepsin-2/3*. Multiple extensions and/or modifications could be made to the PCR panel, depending on the specific use cases. For example, more of the *crt* gene could be included in the multiplex assay, given that variation along this gene has been associated with emerging partner drug resistance in Southeast Asia^10,109^; or adding *hrp2/3* targets to monitor for deletions. Future work can assess assay performance over a wider range of sample types including from asymptomatic and unselected low-parasitaemia cases. We note that all genomic inferences of antimicrobial susceptibility carry a risk of failing to detect phenotypic resistance or predicting resistance that would not manifest in vivo in a given individual. Linked phenotypic data remain essential to ensure that genetic markers are informative in specific populations.

As genomics becomes increasingly decentralized, there is greater need for scientific consensus on best practices for conducting malaria molecular surveillance, quality assurance and processes for open-access data sharing to ensure that locally produced data can be integrated into larger analyses^110^. This will increase the breadth and depth of global malaria surveillance in the drive towards elimination.

## Methods

### Study setting

The study was based at two sites in Ghana with contrasting epidemiology: Ledzokuku Krowor Municipal Assembly (LEKMA) Hospital in Accra, and two satellite clinics in and around Navrongo and the War Memorial Hospital (WMH), in the Upper East Region near the northern border with Burkina Faso. LEKMA Hospital is in an urban setting near the coast where malaria is perennial, and represents a substantial burden of both inpatient and outpatient visits. Navrongo is a more rural setting, situated in a scrub–savannah ecological setting where malaria is strongly seasonal, with high transmission during the rainy season occurring around July–November. Sample collection for the venous blood samples took place from August to September 2022 at both sites, so during the Navrongo high season. Sample collection for dried blood spots took place in Navrongo in 2018.

### Clinical sample collection and processing

This study incorporates samples collected under the governance of two separate studies. Samples from LEKMA Hospital were collected via the Emerging Genomic Selection and Antimalarial Drug Tolerance (EGSAT) study. Samples from Navrongo were collected via the Pan-African Malaria Genetic Epidemiology Network (PAMGEN) study. Both studies had approval from ethical review boards for malaria parasite genomic sequencing research. In both sites, patients presenting with symptoms compatible with malaria were tested using RDTs (OnSite Malaria Pf/Pan Ag Rapid Test by CTKBiotech, reference: R0113C). People positive for at least one of the Pf-specific antigen band (*hrp2/3*) and/or the pan-*Plasmodium* antigen band (LDH) were recruited with informed consent from the patient or their guardian. Around 2–5 ml venous blood samples were collected, of which 0.5–4 ml was typically available to use in this study.

Samples were transported daily, from Monday to Friday, from LEKMA Hospital to the West African Centre for Cell Biology of Infectious Pathogens (WACCBIP), University of Ghana, and from the three Navrongo sites to the Navrongo Health Research Centre (NHRC) Research lab in Navrongo. Leucodepletion was performed by removing the buffy coat layer following centrifugation, using the following steps: blood samples were centrifuged in the EDTA tubes they were collected in at 500 *g* for 5 min with no break, the plasma and any visible buffy layer were carefully removed, an approximately equal volume of PBS was added, the blood sample was spun again under the same conditions, and PBS and any further visible buffy coat plus the thinnest top layer of RBCs were again aspirated (to maximize WBC removal). PBS was added to a final volume of 1–2 ml, and samples were transferred to 15 ml falcon tubes and frozen in the −80 freezer until DNA extraction.

Samples from Navrongo included a prospective collection of leucodepleted VB (collected in 2022) and a retrospective selection of DBS samples, originally collected in 2018. The 87 DBS samples were selected from a larger collection that had already passed MalariaGEN quality control (QC) filtering for Illumina whole-genome sequencing, with the added requirements for parasitaemia to be microscopy positive, that is, ≥1 parasite per 200 WBC by thick film microscopy and DNA concentration post-extraction to be >1 ng μl^−1^.

### Mock clinical samples

Mock DBS samples were produced by combining human whole blood ordered from Cambridge Bioscience Ltd with RBCs infected with *P. falciparum* (Dd2 clone) cultured in vitro and blotting 50 µl onto Whatman 3M cards. *P. falciparum* in vitro culture was performed at the Wellcome Sanger Institute (WSI) as described in ref. ^111^. Final haematocrit of the cultured parasite–whole-blood mixtures was 35%. The volume of parasitized RBCs added to human whole blood was varied to produce an approximate final parasitaemia of 10%, 1%, 0.1% and 0.01% infected RBCs. The expected linear relationship between parasitaemia and the ratio of parasite to human DNA present in the mock DBS samples following DNA extraction was confirmed by qPCR using probes targeting conserved regions of the *P. falciparum* and human genomes (Supplementary Notes).

### DNA extraction and quantification

Four methods for DNA extraction were used. For 87/109 of the prospectively collected VB samples, DNA extraction was performed using the New England Labs Monarch High Molecular Weight (HMW) DNA extraction kit for cells and blood (T3050) according to manufacturer protocol. Twenty-two of the 109 prospectively collected VB samples were extracted using the QIAmp DNA blood mini kit (51106) according to manufacturer instructions with minor modifications detailed in Supplementary Notes. For the mock DBS samples, DNA extraction was performed using the QIAmp DNA investigator kit (56504), and the protocol was adapted from the ‘Isolation of Total DNA from FTA and Guthrie Cards’ with minor modifications detailed in Supplementary Notes. Finally, for the clinical DBS samples, DBS samples were transferred from Ghana to the WSI and DNA was extracted using the QIAamp Investigator Biorobot kit on the Qiagen Biorobot Universal instrument using a custom protocol described in Supplementary Notes.

DNA was quantified using a Qubit 2.0 fluorometer (ThermoFisher) with Qubit dsDNA high-sensitivity (Q32854) and Qubit dsDNA broad-range kits (Q32853) following manufacturer instructions.

### Primer design and PCR amplification

Primers were designed using the ‘primer3’ software^112–114^. Primer regions were selected on the basis of sequence conservation after aligning target genes in *P. falciparum* from the reference genomes produced in ref. ^115^. Primer compatibility for multiplexing was assessed in silico using the ThermoFisher Multiple Primer Analyzer (https://www.thermofisher.com/de/de/home/brands/thermo-scientific/molecular-biology/molecular-biology-learning-center/molecular-biology-resource-library/thermo-scientific-web-tools/multiple-primer-analyzer.html). Multiple iterations of primer combinations were tested and assessed by gel electrophoresis to identify the most robust combinations (producing the brightest bands down to the lowest parasitaemias with mock clinical DBS and with minimal non-specific bands). Multiple iterations of PCR optimization were undertaken to yield the final reaction conditions used.

All of the samples described in this study underwent multiplex drug resistance and *csp* amplification using Platinum *Pfx* DNA polymerase (ThermoFisher, 11708039), with reaction conditions shown in Supplementary Notes. The Platinum *Pfx* DNA polymerase enzyme has been discontinued by the manufacturer. We found that the Kapa HiFi polymerase produces comparable results using the same primers. The *msp1* PCR was performed using Promega long-range Go*Taq* polymerase (M4021), with reaction conditions described in Supplementary Notes.

After PCR, a subset of samples from each 96-well plate, always including the positive and negative controls for that plate, were inspected by gel electrophoresis to ensure that the PCRs had been successful (with blank negative controls) before proceeding to nanopore sequencing. A volume of 2–4 µl of the drug resistance and *csp* multiplex PCR was run for 45–90 min on a 2% agarose gel at 100 V. A volume of 2–4 µl of the *msp1* PCR was run for 45–90 min on a 1% agarose gel at 100 V. PCRs were extracted and purified using the Qiagen MinElute PCR purification kit (28004). The full volumes of both the multiplex and *msp1* PCRs for each sample were combined at this stage, each being added to the same extraction column such that each sample yielded a single eluted solution including both PCR reactions. Samples were eluted in 100 µl elution buffer. Post-extraction DNA quantification was performed using the Qubit fluorometer as described above.

### Nanopore library preparation and sequencing

For the prospective leucodepleted VB samples (*n* = 109), library preparation was carried out using ONT kit SQK-NBD112.24 following the ‘ligation sequencing amplicons–native barcoding’ protocol. Manufacturer instructions were followed, except that Blunt/TA ligase master mix was substituted with NEB Quick T4 DNA ligase and NEBNext Quick Ligation reaction buffer (5X) in the ‘native barcoding ligation’ (step 5) for three of the clinical sample libraries (labelled VB D, E and F) due to depletion of the Blunt/TA ligase master mix during field work without ready access to replacements. We did not observe any drop in yield for the libraries that used NEB Quick T4 DNA ligase compared with Blunt/TA ligase master mix. Additional nuclease-free water was added to ensure a final volume of 20 µl. For the negative controls, nuclease-free water was added to the same PCR reaction mixes, which were then taken through the full workflow including PCR, extraction and nanopore library prep. Five batches of 24 and one of 15 samples were sequenced in six MinION runs, each with a fresh R10.4 flow cell; this included technical replicates for internal quality assessment. The MinION runs with VB samples are referred to by the letters VB A–F in the main text. Every run included 1 positive and 1 negative control.

For the retrospectively sequenced batch of DBS samples (*n* = 87), sequencing was performed using the ONT kit SQK-NBD114.24 following the ‘ligation sequencing amplicons–native barcoding’ protocol following manufacturer instructions. Four batches of 24 samples were sequenced in four MinION runs, each with a fresh R10.4.1 flow cell (referred to as DBS A–D); as with the VB samples, each run comprised 22 clinical samples, a negative control and a positive control. A single sample went through PCR and sequencing twice to assess for reproducibility of results. The ‘validation’ sample set of laboratory isolates and mock DBS samples was sequenced both with Q20 chemistry (kit SQK-NBD112.24, R10.4 flow cells) and with Q20+ chemistry (kit SQK-NBD114.24, R10.4.1 flow cells) at 400 bps.

Sequencing and real-time base calling using the kit 12/R10.4 flow cells was performed using the MinKNOW software v.22.05.5 (Bream 7.1.3, Configuration 5.1.5, Guppy 6.1.5, MinKNOW Core 5.1.0). Sequencing and real-time base calling with the kit 14/R10.4.1 flow cells was performed using MinKNOW v.22.10.10 (Bream 7.3.5, Configuration 5.3.8, Guppy 6.3.9, MinKNOW Core 5.3.1).

### Hardware and workstation set-up

Sequencing, base calling and the real-time bioinformatic analysis were run from a commercial Dell gaming laptop with the following specifications: 11th Gen Intel Core Processor i7 (8 Core); 32 GB (2x 16 GB) DDR4, 3,200 MHz; GPU: NVIDIA GeForce RTX 3080 with 16 GB GDDR6; 1 TB M.2 solid state drive. During nanopore sequencing, the laptop was connected to an uninterruptible power supply with surge protection. Additional fans were used to reduce laptop and MinION mk1b device overheating.

### Bioinformatics

#### Real-time base calling and analysis using the ‘nano-rave’ Nextflow pipeline

Base calling was done in real-time alongside sequencing using the MinKNOW software. We tested both high-accuracy (HAC) and super-accurate (SUP) ‘guppy’ base calling runs via the laptop’s GPU. Analyses included in this study for the clinical samples were performed on SUP base-called reads. The resulting fastq files were processed through a custom Nextflow pipeline: nano-rave (Nanopore Rapid Analysis and Variant Explorer), run on the laptop using Debian as a Linux operating system for Windows. The nano-rave pipeline is available via GitHub at: https://github.com/sanger-pathogens/nano-rave. Briefly, following QC metrics, sequence reads were mapped against 3D7 reference sequences for each of the amplicon target genes using minimap2 (ref. ^116^). Mapping to individual reference sequences for target genes, rather than to the whole genome, substantially reduces computational requirements for the workflow, allowing it to run at speed directly on a commercial laptop. .sam files were converted to .bam files using samtools^117^. Amplicon coverage data were generated using ‘BEDTools’^118^. There are several parameterized options available for variant calling: ‘medaka variant’, ‘medaka haploid’^78^ and ‘freebayes’^79^. (Subsequently, ‘Clair3’ has been added.) These generate variant call format (VCF) file outputs for each amplicon for each sample (ONT barcode). We tested ‘medaka variant’ and ‘medaka haploid’ on all clinical samples and used ‘medaka haploid’ genotypes for downstream analyses described in the main text. VCF files were processed using custom R scripts to calculate SNP allele frequencies at key drug resistance loci. A cut-off of >50× coverage was applied for an amplicon to be included in the analysis; however, all amplicons for all samples in the study exceeded this cut-off. None of the negative controls included in this study generated directories that were >10 MB in size, which was used as a parameterized cut-off in the nano-rave workflow; therefore, no negative controls were taken forward for real-time analysis.

#### Whole-genome mapping and manual inspection

In addition to the real-time analysis performed on the laptop in Ghana outlined above, SUP base-called reads were mapped genome-wide to the 3D7 reference genome using ‘minimap2’ on the WSI high performance computing cluster. Read pile-ups for each amplicon locus were manually inspected using the IGV tool^119^.

#### Consensus sequence generation for *csp* and *msp1*

Consensus sequences for *csp* and *msp1* were produced for the laboratory clones using ‘Amplicon_sorter’^83^, a tool for building reference-free consensus sequences using ONT-sequenced amplicons based on read similarity and length. Reads mapping to the 3D7 *csp* reference sequence were extracted and used as input for ‘Amplicon_sorter’ using a similarity cut-off of 96% (the threshold for merging sequences to generate consensus). For *msp1*, reads in the expected size range (~4,700–5,300 bp) were pulled directly from the fastq files for consensus sequence building. For single laboratory clones, a threshold of 96% was used for consensus merging. For mixed isolates, this was increased to 98% to distinguish between the reference isolates used. The resulting consensus sequences were trimmed to include only the sequences within the primer sites and reverse complemented if needed. Consensus sequences were then mapped against the expected reference sequence using the Clustal Omega online tool.

### Ethical approvals

The Navrongo samples were collected as part of the PAMGEN study, with ethics approval ID: NHRCIRB343, obtained from the NHRC Institutional Review Board. This includes both the prospectively sequenced leucodepleted VB samples (collected in 2022) and the DBS samples (collected in 2018). The LEKMA Hospital samples (collected in 2022) were collected as part of the EGSAT study, ethics ID: ECBAS030/21–22, approved by the College of Basic and Applied Sciences Ethics Review Committee, University of Ghana. All participants or their guardians (as appropriate) were provided detailed information sheets and gave informed consent before enrollment. Further approval was granted by the WSI’s Research Ethics Committee for the analysis of the samples. All patient-identifiable data are securely stored by L.N.A.-E. and only non-patient-identifiable data were provided to the WSI. The study complies with all relevant ethical regulations.

### Inclusion and ethics statement

This research was conducted as a collaboration between researchers based at the University of Ghana, the NHRC and the WSI, United Kingdom. All stages of the research including conceptualization, study design, sample collection, sequencing, data analysis, data visualization and authorship of publications were conducted collaboratively between personnel based in Ghana and the United Kingdom. The research is highly locally relevant to Ghana, where malaria continues to pose a major threat to public health, and builds on ongoing malaria surveillance work in Ghana led by co-lead author L.N.A.-E. The project included activities to strengthen nanopore sequencing capacity at the University of Ghana and the NHRC.

### Reporting summary

Further information on research design is available in the Nature Portfolio Reporting Summary linked to this article.

### Supplementary information

Supplementary Information Supplementary Tables 1–5 and Notes.

Reporting Summary

Supplementary Table 6 Sample metadata with ENA accession codes.

## Data Availability

*P. falciparum* nanopore amplicon sequence data, with human genetic data removed, can be accessed from the ENA via study accession ERP145278, with sample metadata available in Supplementary Table 6. The *P. falciparum* reference sequences used were from the 3D7 v3.0 reference genome, accessed from PlasmoDB.

## Code availability

The nano-rave pipeline for processing base-called nanopore sequence data (fastq file input) to produce quality control metrics, coverage data and variant calls (vcf files) is available via GitHub at https://github.com/sanger-pathogens/nano-rave.
