# Supplementary material for: Drug resistance and vaccine target surveillance of Plasmodium falciparum using nanopore sequencing in Ghana
Source: Nat Microbiol. 2023 Nov 23;8(12):2365–77. doi: 10.1038/s41564-023-01516-6 (PMC10686832; doi:10.1038/s41564-023-01516-6)
Supplement: Supplementary file 1 — Supplementary Tables 1–5 and Notes. [file 41564_2023_1516_MOESM1_ESM.pdf]

nature microbiology

Article https://doi.org/10.1038/s41564-023-01516-6

Drug resistance and vaccine target surveillance of *Plasmodium falciparum* using nanopore sequencing in Ghana

In the format provided by the authors and unedited

Drug resistance and vaccine target surveillance of *Plasmodium falciparum* using nanopore sequencing in Ghana

Supplementary information

Table of Contents

Supplementary Tables ***2***

Supplementary Table 1 .... **2**

Supplementary Table 2 .... **3**

Supplementary Table 3 .... **4**

Supplementary Table 4 .... **5**

Supplementary Table 5 .... **6**

Supplementary Notes ***7***

Study settings – further information **7**

DNA extraction methods – further information **7**

nano-rave **pipeline and variant calling** **9**

PCR reaction conditions **12**

qPCR to estimate human and *P. falciparum* gDNA relative abundance **14**

Inference rules for drug resistance phenotyping from genotype data **15**

Supplementary Tables

Supplementary Table 1

Sample batch name	ONT chemistry used	Amplicon samples per flow cell (of which clinical samples)	Run time	Data produced (GB)	Estimated bases (Gb)	Reads generated (M)	Bases called (real-time), pass (Gb)
VB A	Kit 12, R10.4	24 (22)	6 hours	33.04	1.66	1.26	1.25
VB B	Kit 12, R10.4	24 (22)	6 hours 40 mins	33.52	1.55	1.71	1.26
VB C	Kit 12, R10.4	15 (13)	6 hours	37.05	1.62	2.08	1.27
VB D	Kit 12, R10.4	24 (22)	8 hours	36.47	1.71	1.77	1.29
VB E	Kit 12, R10.4	24 (22, with 14 repeats)	8 hours	31.96	1.49	1.65	1.19
VB F	Kit 12, R10.4	24 (22)	8 hours	43.31	1.76	2.68	1.34
DBS A	Kit 14, R10.4.1	24 (22)	8 hours	57.36	2.64	4.38	2.1
DBS B	Kit 14, R10.4.1	24 (22)	8 hours	71.94	3.49	5.2	2.88
DBS C	Kit 14, R10.4.1	24 (22)	6 hours	43.07	2.07	3.2	1.8
DBS D	Kit 14, R10.4.1	24 (22, with 1 repeat)	6 hours	54.3	2.51	4.21	2.17
V12	Kit 12, R10.4	17 (0)	6 hours	39.13	1.86	2	1.61
V14	Kit 14, R10.4.1	17 (0)	6 hours	52.11	2.76	3.47	2.57

Nanopore data generation. All sequencing was performed using an ONT MinION mk1b device with real-time base calling using the Super Accurate (SUP) *guppy* option. Samples were multiplexed using ONT native barcoding and sequenced in batches on fresh flow cells. There were six batches of venous blood (VB) samples, denoted VB A – F; and 4 batches of dried blood spot (DBS) samples, denoted DBS A – D. Most batches had 22 clinical samples, except for VB C which had 15; all batches included 1 positive control (gDNA from one of the Dd2, HB3 or KH2 clones) and 1 negative control. Batch VB E included 15 samples that were repeats of samples included in batches VB A – D, to assess for assay consistency. DBS D also included 1 sample repeat. In total, 109 unique venous blood samples and 87 unique dried blood spot samples were sequenced (**Figure 1**). All venous blood samples were sequenced using ONT kit 12, R10.4 flow cells; all dried blood spot samples were sequenced using ONT kit 14, R10.4.1 flow cells (400bps). In addition, a 'validation' (V) set of samples was sequenced, comprising laboratory isolates (3D7, Dd2, HB3, 7G8, GB4, KH1 and KH2 clones), 2 clone mixtures, 8 mock DBS samples in pairs at each parasitaemia of 10%, 1%, 0.1% and 0.01% infected RBCs, and a negative control. The validation set was sequenced both using ONT kit 12, R10.4 flow cells ('V12' batch) and kit 14, R10.4.1 flow cells ('V14' batch, 400bps). The V12 and V14 validation samples were identical. Compared with kit 12/ R10.4, we found that kit 14/ R10.4.1 produced more data with improved pore retention during sequencing.

Supplementary Table 2

DHFR haplotype	Haplotype count	Haplotype frequency	Haplotype %
IRNI	163	0.83163265	83.2
NRNI	17	0.08673469	8.67
NCSI	13	0.06632653	6.63
ICNI	2	0.01020408	1.02
NCNI	1	0.00510204	0.51

DHFR haplotype frequencies. Showing haplotype frequencies (i.e. combinations of majority genotype calls) for the 196 samples analysed in the study (comprising 109 leucodepleted venous blood and 87 dried blood samples).

Supplementary Table 3

**DHPS haplotype**	**Haplotype count**	**Haplotype frequency**	**Haplotype %**
AGKAA	80	0.40816327	40.8
SGKAA	73	0.37244898	37.2
AGKAS	20	0.10204082	10.2
AAKAA	11	0.05612245	5.61
SAKAA	5	0.0255102	2.55
AGKGS	4	0.02040816	2.04
SAKAS	3	0.01530612	1.53

DHPS haplotype frequencies. Showing haplotype frequencies (i.e. combinations of majority genotype calls) for the 196 samples analysed in the study (comprising 109 leucodepleted venous blood and 87 dried blood samples).

Supplementary Table 4

DHFR + DHPS haplotype	Haplotype count	Haplotype frequency	Haplotype %
dhfr-IRNI, dhps-AGKAA	70	0.35714286	35.7
dhfr-IRNI, dhps-SGKAA	60	0.30612245	30.6
dhfr-IRNI, dhps-AGKAS	19	0.09693878	9.69
dhfr-NRNI, dhps-AGKAA	7	0.03571429	3.57
dhfr-IRNI, dhps-AAKAA	6	0.03061224	3.06
dhfr-NRNI, dhps-SGKAA	6	0.03061224	3.06
dhfr-NCSI, dhps-SGKAA	5	0.0255102	2.55
dhfr-IRNI, dhps-SAKAA	4	0.02040816	2.04
dhfr-NRNI, dhps-AAKAA	4	0.02040816	2.04
dhfr-NCSI, dhps-AGKAA	3	0.01530612	1.53
dhfr-IRNI, dhps-AGKGS	2	0.01020408	1.02
dhfr-IRNI, dhps-SAKAS	2	0.01020408	1.02
dhfr-NCSI, dhps-AGKGS	2	0.01020408	1.02
dhfr-ICNI, dhps-AAKAA	1	0.00510204	0.51
dhfr-ICNI, dhps-SGKAA	1	0.00510204	0.51
dhfr-NCNI, dhps-SGKAA	1	0.00510204	0.51
dhfr-NCSI, dhps-AGKAS	1	0.00510204	0.51
dhfr-NCSI, dhps-SAKAA	1	0.00510204	0.51
dhfr-NCSI, dhps-SAKAS	1	0.00510204	0.51

DHFR + DHPS combined haplotype frequencies. Showing haplotype frequencies (i.e. combinations of majority genotype calls) for the 196 samples analysed in the study (comprising 109 leucodepleted venous blood and 87 dried blood samples).

Supplementary Table 5

SNP	Count	Frequency (%)
D288N	1	0.51
D294N	6	3.06
E295K	1	0.51
N298K	24	12.2
A299G	4	2.04
S301N	176	89.8
A302G	1	0.51
K314Q	82	41.8
K317E	164	83.7
K317T	7	3.57
E318K	88	44.9
E318Q	72	36.7
L320I	1	0.51
N321K	148	75.5
N321Q	12	6.12
K322I	30	15.3
K322R	12	6.12
K322T	77	39.3
Q324K	74	37.8
Q324R	8	4.08
L327I	18	9.18
N352D	8	4.08
P354S	7	3.57
K355R	1	0.51
D356E	1	0.51
D356N	12	6.12
E357Q	125	63.8
D359N	30	15.3
A361E	94	48

List of Single Nucleotide Polymorphisms (SNPs) in the C-Terminal Region (CTR) of circumsporozoite protein (CSP). Ordered by amino acid position of discovered variants. Note the C-terminal domain in 3D7 can be considered as spanning amino acid positions 273 to 397. Nomenclature is based on differences from the amino acid position for the 3D7 reference sequence.

Supplementary Notes

Study settings – further information

The study was based at two sites in Ghana with contrasting epidemiology: Ledzokuku Krowor Municipal Assembly (LEKMA) Hospital in Accra, and clinics based in and around Navrongo in the Upper East Region near the northern border with Burkina Faso. Sample collection in Navrongo took place at three sites: The Navrongo War Memorial Hospital (WMH) and Navrongo Central Clinic (NCC), within Navrongo town, and Biu Health Centre (BHC), around 30 km southwest of Navrongo. The NCC and BHC are community clinics, while WMH is the Municipal hospital with facilities for inpatient care. The Navrongo samples thus reflect community mild malaria cases that are RDT positive (baseline parasitaemia data for the region presented in **Extended data Figure 5**). Samples from LEKMA hospital may represent a more selected patient group – both increased hospitalisation and prioritisation of which samples were collected from LEKMA may bias the cohort to higher parasitaemias, so direct comparison of parasitaemias between the Navrongo and LEKMA sites is confounded. Prospective collection of venous blood samples took place in Navrongo and Accra August – September 2022 (with a minority of samples in Accra also collected April – July 2022). A set of dried blood spots (DBS) were collected in Navrongo in 2018 and included for retrospective analysis.

DNA extraction methods – further information

Four methods for DNA extraction were used. For 87/109 of the prospectively collected venous blood samples, DNA extraction was performed using the New England Labs Monarch® High Molecular Weight (HMW) DNA Extraction Kit for Cells & Blood (T3050) according to the manufacture's protocol. Part 1: erythrocyte lysis was conducted on frozen samples (-80°C) in 15 ml falcons with ~2 ml sample volume. After centrifugation, ~4-5 ml of supernatant was discarded. The pellet was dislodged, vortexed and transferred to a 2 ml Lo-Bind Eppendorf tube. A minimum of two 1xPBS washes were required and subsequent washes were carried out until the supernatant was clear. Part 2: leukocyte lysis: standard input volumes were required for all steps. Part 3: HMW gDNA binding and elution: isopropanol standard input volume; DNA was generally eluted in 110µl Elution Buffer (EB), though for a small number of samples where a large DNA pellet was visible, 210µl EB was used. Minor modifications were made to the protocol due to equipment availability; 1) samples were not kept on ice 2) all centrifuge steps were conducted at room temperature 3) samples underwent manual rotation as opposed to using a vertical rotating mixer 4) a heat-block replaced the incubator.

22/109 of the prospectively collected venous blood samples were extracted using the QIAmp® DNA Blood Mini Kit (51106) according to manufactures instructions with the following modifications: 1) 200 µl of frozen washed RBCs were thawed, and Protease was substituted with proteinase K. 2) Samples were incubated for 56°C for 30 minutes. 3) Samples were transferred into spin columns and centrifuged at

8000 rpm for 1 minute and 30 seconds. 4) Buffer AW1 was added and centrifuged at 8000 rpm for 1 minute and 30 seconds. 5) Buffer AW2 was added and centrifuged for at 13000 rpm for 3 minutes. 6) An elution volume of 100µl was added to the spin columns and incubated at room temperature for 15 minutes. Extracted DNA was stored at -20°C.

For the mock DBS samples, DNA extraction was performed using the QIAmp DNA Investigator Kit (56504), and the protocol was adapted from the 'Isolation of Total DNA from FTA and Guthrie Cards'. Modifications to the protocol included adjusted quantities and an overnight incubation step: 1) 6 (1/8 inch) diameter punches placed into a 15 ml falcon tube, 2) 600 µl of Buffer ATL, 3) 60 µl of proteinase K, 4) Incubation at 56°C with shaking at ~600rpm for ~17 hours, 5) 600 µl of Buffer AL. Final steps were also modified to increase DNA concentration; 1) adjusted elution volume of 50 µl 2) spin column incubated for 5 minutes at room temperature 3) once centrifuged, eluate placed back into the spin column followed by a 5 minute room temperature incubation.

For the clinical DBS samples, DBS were transferred from Ghana to the Wellcome Sanger Institute. DNA was extracted using the QIAamp Investigator Biorobot kit on the Qiagen Biorobot Universal instrument using a custom protocol. For each DBS, 7 x 4mm discs were punched per well of a deepwell plate (puncher used: BSD600 Plus, BSD Robotics). A mastermix of buffer ATL and proteinase K was made and 660ul added per well (600ul ATL, 60ul proteinase K). The plates were incubated overnight at 56C for 17h, with shaking at 600rpm (Bioshake iQ, Q instruments). 600ul of lysate was transferred into a Qiagen S-block, which was loaded onto the Qiagen Biorobot Universal for extraction. On the Biorobot, a series of bind, wash and elution steps are performed. DNA is absorbed onto a silica membrane, and an integrated vacuum system draws lysates and buffers through, prior to eluting in 100ul nuclease free water. Key process modifications involved scaling up reaction volumes and increasing the elution incubation time to maximise DNA yield. Wash buffer volumes were increased accordingly, to minimise carryover of blood into the final eluate.

nano-rave pipeline and variant calling

An outline of the *nano-rave* pipeline is shown below:

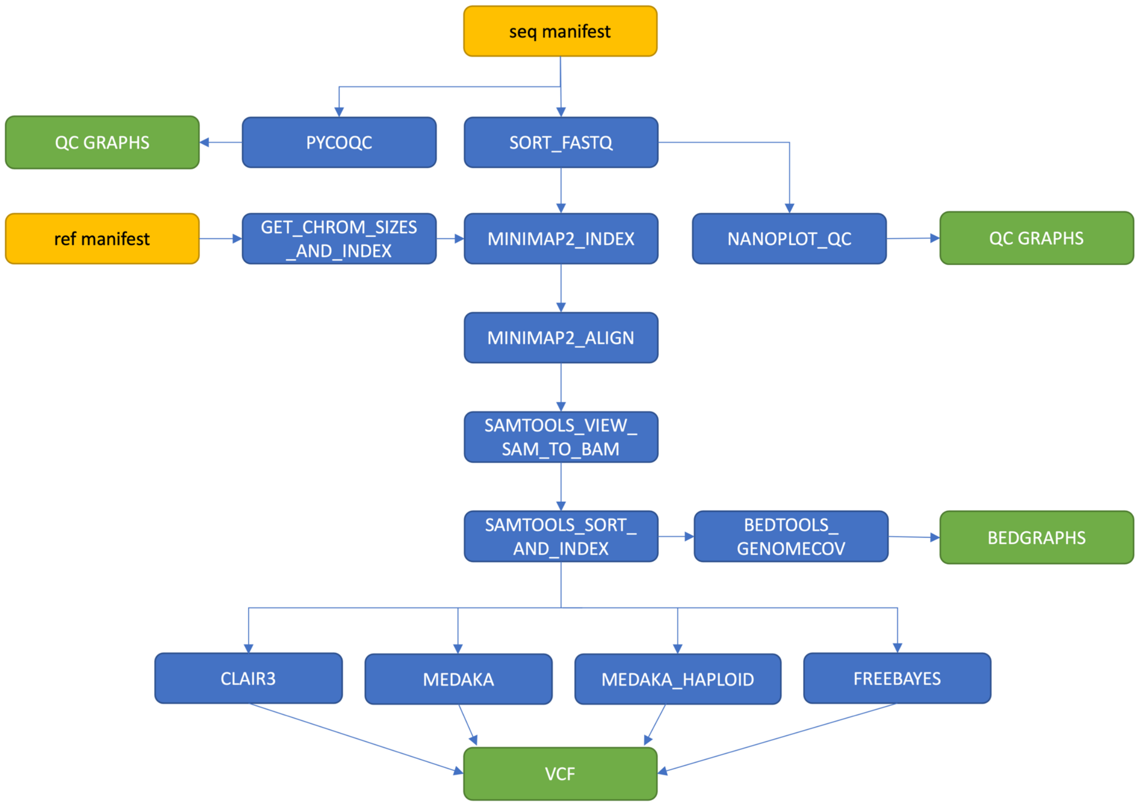

Nano-rave is run through the terminal. Full details of the pipeline including installation and user guides can be found at: https://github.com/sanger-pathogens/nano-rave.

We used *nano-rave* to align sequence reads against each amplicon reference (3D7) sequence, rather than against the full *P. falciparum* genome, to reduce computation requirements – although, this is not inherent to the pipeline and whole genome alignments could be performed. Several variant callers are available in the pipeline to produce VCF files. *Freebayes* is a Bayesian genetic variant detector designed for short read sequence data. *Medaka* uses neural networks to create consensus sequences and variant calls from Nanopore data. *Medaka_variant* was designed for diploid genomes and relatively lower coverage whole genome sequence data, and is being replaced by newer algorithms such as *Clair3*. *medaka_haploid_variant* was designed for calling variants in monoploid organisms, based on comparing the consensus sequence built in *medaka* against a reference sequence. We felt that *medaka_haploid_variant* (referred to as *medaka haploid*) was well suited to generating majority genotype calls from the consensus sequence for each amplicon; 'heterozygous' positions (reflecting mixed infections) would therefore be genotyped by the majority allele present in that infection at that position. Subsequently, *Clair3* has been added to *nano-rave* as another available variant caller.

The *nano-rave* pipeline currently uses the following software (versions in brackets): bedtools (2.29.2), clair3 (1.0.0), freebayes (1.3.5), medaka (1.4.4), minimap2 (2.17), nanoplot (1.38.0), pycoqc (2.5.2), samtools (1.15.1), tabix (1.11).

An example *nano-rav*e command using the *medaka-haploid* variant caller is shown below:

```
nextflow run main.nf --sequencing_manifest /path_to_seq_manifests/seq_manifest_sampleA.csv
--reference_manifest /path_to_ref_manifests/ref_manifest_DRAG1.csv --results_dir /path_to_results
-with-trace --variant_caller medaka_haploid
```

An example *nano-rave* command using *Clair3* for variant calling is shown below:

```
nextflow run main.nf --sequencing_manifest /path_to_seq_manifests/seq_manifest_sampleA.csv
--reference_manifest /path_to_ref_manifests/ref_manifest_DRAG1.csv --variant_caller clair3
--clair3_args "--model_path /opt/models/r941_prom_sup_g5014 --no_phasing_for_fa
--include_all_ctgs" --results_dir /path_to_results -with-trace
```

The drug resistance SNPs genotyped using the *nano-rave* workflow with *medaka haploid* matched the expected positions for the reference isolates tested (3D7, Dd2, HB3, 7G8, GB4, KH1 and KH2). Aligned reads were also manually inspected using IGV. The clone mixtures tested in the validation set (3D7 and KH2 clones in 80:20 or 20:80 ratios) confirmed this approach yielded the expected results, ie. genotypes were based on the majority clone in the mixture (see main text). We note that using this method, each locus strictly must be treated in isolation, making haplotype inference unreliable. This is because, depending on the proportion of clones present and their individual genotypes, the majority at one locus does not necessarily belong to the same haplotype as the majority at a different locus. Haplotype inference from deconvoluted mixed infections using nanopore reads is an important area for future research. However, the majority SNP genotyping method applied here can nonetheless produce information useful for malaria surveillance rapidly, such as the prevalence of specific markers of antimalarial drug resistance.

On our laptop, the workflow for a multiplexed batch of 15-24 samples using *medaka variant* and *medaka haploid* variant calling took around 16-23 minutes, depending on the number of samples, amplicons and depth of coverage; *medaka haploid* tended to be slightly faster than *medaka variant*. *Freebayes* took greater than 45 minutes per multiplexed batch and so was not used further for real-time analysis of clinical samples. For 15 samples, the workflow from PCR to sequencing and variant calling was repeated to assess for assay consistency, using both *medaka variant* and *medaka haploid*. Using *medaka variant*, two discrepancies in drug resistance marker SNPs were identified. Both were samples with mixed infections in the discordant SNP as indicated by manual read inspection in IGV, with each sample in the discordant pair being assigned a different allele from the mixture in the two repeats. No discrepancies were identified in this sample set using *medaka haploid* genotype calls, and this variant caller was used for the downstream analyses described in main text.

We observed some inconsistency between sequencing repeats for the Dd2 clone in how position 86 of *mdr1* was genotyped using both *medaka variant* and *medaka haploid*. The wild-type (3D7 sequence) is 86-N, encoded by AAT; Dd2 contains both 86-Y and 86-F, encoded by TAT and TTT, respectively, in different copies of this gene within its genome. One of the mock DBS samples genotyped 86-F here, while the 7 mock DBS samples were 86-Y. Inspection of the read pile-ups in IGV suggested an approximately 50:50 ratio of these two SNPs, with 7 clones having slightly higher frequencies for 86-Y and the one 86-F clone having a slightly higher proportion of that SNP. This likely reflects chance variation in the workflow from PCR amplification to sequencing. Thus, there is potential for mixed infections (or heterozygosity in multi-copy genes, as in *mdr1*) to produce

discrepancies in majority genotype calls, particularly if the proportion of each clone is similar and/or if the starting amount of parasite DNA is very low, as the effect of chance differences in PCR amplification during early cycles may be exaggerated. We note that this problem would also apply to Illumina-based amplicon sequencing methods.

PCR reaction conditions

Primer sequences for the combined drug resistance and *csp* PCR are shown below:

Primer name	Primer sequence
crt F	TGTCTTGGTAAATGTGCTCA
crt R	AGTTGTGAGTTTCGGATGTT
dhfr F	GTTTTCGATATTTATGCCATATGTG
dhfr R	TGATAAACAACGGAACCTCC
dhps F	TTTGTTGAACCTAAACGTGC
dhps R	AACATTTTGATCATTCATGCAAT
mdr1 F	TGTGTTTGGTGTAATATTAAAGAACA
mdr1 R	ACATAAAGTCAAACGTGCATTT
kelch13 F	AAGCCTTGTTGAAAGAAGCA
kelch13 R	GGGAACTAATAAAGATGGGCC
csp F	TGGGAAACAGGAAAATTGGTAT
csp R	TACGACATTAAACACACTGGAA

Primer sequences for the separate *msp1* PCR are shown below:

Primer name	Primer sequence
msp1 F	AGAAGATGCAGTATTGACAGGT
msp1 R	GAACTGCAGAAAATACCATCGA

The position of each amplicon within the respective target gene sequence, relative to 3D7 reference, is shown below:

gene	Amplicon start	Amplicon end	Amplicon size
crt	118	295	177
dhfr	22	512	490
dhps	1222	1863	641
mdr1	228	591	363
kelch13	1277	2145	868
csp	168	1143	975
msp1	105	5099	4994

The reaction mixture for the combined drug resistance and *csp* PCR used for leucodepeleted venous blood samples is shown below for a single sample (these values would be multiplied accordingly to prepare master mixes). The concentration of gDNA used was typically 5-10ng/ul.

Reagent	1x for 50ul
10x Buffer	6
10mM dNTP mix	2.25
MGSO4 (50mM)	1.5
Primer (50µM) - crt F	0.5
Primer (50µM) - crt R	0.5

Primer (50µM) - dhfr F	0.6
Primer (50µM) - dhfr R	0.6
Primer (50µM) - dhps F	1
Primer (50µM) - dhps R	1
Primer (50µM) - mdr1 F	0.6
Primer (50µM) - mdr1 R	0.6
Primer (50µM) - k13 F	0.8
Primer (50µM) - k13 R	0.8
Primer (50µM) - csp F	1
Primer (50µM) - csp R	1
DNA Pol	0.5
gDNA	4
H2O	26.75
Total	50

All of the samples described in this study underwent multiplex drug resistance and *csp* amplification using the Thermo Fisher Platinum™ *Pfx* DNA Polymerase (11708039). Note that the quantity of primers added for each reaction varied according to the reaction efficiency, to attempt to make amplicon coverage more even. The Platinum™ *Pfx* DNA Polymerase enzyme has been discontinued by the manufacturer. We have found that the Kapa HiFi polymerase (KK2101, manufacturer conditions) produces comparable results using the same primers.

The reaction conditions for the drug resistance and *csp* multiplex PCR using *Pfx* DNA Polymerase, used for leucodepeleted venous blood samples, are shown below:

**Step no.**	**Step**	**Temp**	**Duration**
1	Initial denature	94	5 min
2	Denature	94	15 sec
3	Anneal	54	30 sec
4	Extend	68	1 min
5	Repeat steps 2-4 34x more times		
6	Store	4	Forever

The drug resistance and *csp* multiplex PCR with *Pfx* DNA Polymerase used for dried blood spot (DBS) samples was the same as that used for leucodepleted venous blood samples (above) except for the following modifications: 15ul of extracted gDNA was used as template for the PCR reaction (with accordingly reduced H2O added to reach 50ul reaction total), and 40 PCR cycles was used instead of 35 cycles (i.e. step 5 was repeated 39 more times instead of 34).

The reaction mix for the *msp1* PCR is shown below:

	**1x**
GoTaq MM	25

msp1 F (10uM)	1
msp1 R (10uM)	1
gDNA	2
H2O	21
Total	50

The reaction conditions for the *msp1* PCR are shown below:

Step no.	Step	Temp	Duration
1	Initial denature	95	2 min
2	Denature	93	20 sec
3	Anneal	54	25 sec
4	Extend	65	5 min
5	Repeat steps 2-4 34x more times		
6	Final extend	72	10 min
7	Store	4	Forever

The *msp1* PCR was performed using Promega long-range GoTaq® Polymerase (M4021).

qPCR to estimate human and *P. falciparum* gDNA relative abundance

Quantitative PCR (qPCR) was used to assess the ratio of *P. falciparum* to human DNA present in the mock clinical DBS samples and the clinical DBS samples collected in Navrongo. Following DNA extraction, qPCR was performed on a Roche LightCycler®480 Instrument II with a fluorescent probe method using distinct dyes FAM, HEX and RED 640. The primer/probes from Integrated DNA Technologies (IDT) were designed on PrimerBLAST; AMA1 for *P. falciparum*, PVX_096055 for *Plasmodium vivax* (VIV3) and PDGFRB for *Homo sapiens* (PLAT1). Probe dyes; FAM for VIV3, LightCycler 640 for AMA1 and HEX for PLAT1. The addition of an internal quencher, ZEN was used for both HEX and FAM dyes. The *P. vivax* probe played no role in this study. Primer sequences are shown below.

Name	Type	Primer sequence
VIV3	Primer-forward	AAA GAT TCG TAG CTG TCG GTG GGT
	Primer-reverse	TTC CAT TAA GTG CGC GTA CCG AGA
	Probe	ACA GCG ACG ACT CCA GAT CCG ATT TA
AMA1	Primer-forward	TGC CAT ATA TTC CGT CCA TGG
	Primer-reverse	ACG CAT ATC CAA TAG ACC ACG
	Probe	CGA ACC CGC ACC ACA AGA ACA AAA
PLAT1	Primer-forward	CTT ACC ACA TCC GCT CCA TC
	Primer-reverse	TTC ACA CTC TCC GTC ACA TTG
	Probe	CAC ATC CCC AGT GCC GAG TTA GA

A master mix was prepared with the following components (quantities provided per well); 10µl Roche LightCycler®480 Probes Master (04887301001), 6µl Nuclease-free water (AM9937) 1µl AMA1 Primer/probe, 1µl PLAT1 Primer/probe, and 1µl VIV3 Primer/probe. Sample volumes: 1µl sample DNA, 1 µl negative control and 1 µl positive controls (standards), with all samples including a repeat.

Program selection was ‘Abs quant’. PCR conditions are shown below:

Step	Temperature	Duration	Cycles
1	94°C	5 minutes	
2	57°C	20 seconds	Steps 2-4: Total of 45 cycles
3	72°C	1 second	
4	95°C	10 seconds	
5	40°C	infinite	

Inference rules for drug resistance phenotyping from genotype data

Inference rules were based on Jacob, C., *et al*. 2021. *eLife* 10:e62997, summarised below:

**Antimalarial drug**	**Gene**	**Mutation**	**Interpretation**
Chloroquine (CQ)	*crt*	76-K<br>76-T	Susceptible<br>Resistant
Pyrimethamine (PYR)	*dhfr*	108-S<br>108-N	Susceptible<br>Resistant
Sulfadoxine (SX)	*dhps*	437-A<br>437-G	Susceptible<br>Resistant
Sulfadoxine-Pyrimethamine (SP) for malaria treatment	*dhfr*	51-N or 59-C or 108-S<br>51-I & 59-R & 108-N	Susceptible<br>Resistant
Sulfadoxine-Pyrimethamine (SP) for IPTp	*dhfr* & *dhps*	*dhfr*: 51-N or 59-C or 108-S<br>& (*dhps*: 437-A or 540-K)<br>& (*dhfr*-164-I or *dhps*-581-A or *dhps*-613-A)<br><br>*dhfr*: 51-N & 59-C & 108-S<br>& *dhps*: 437-G & 540-E<br>& (*dhfr*-164-L or *dhps*-581-G or *dhps*-613-(S/T))	Susceptible<br><br><br><br>Resistant
Artemisinin	*kelch13*	Mutations associated with artemisinin partial resistance*	Artemisinin partial resistance

Crt = chloroquine resistance transporter, PF3D7_0709000; *dhfr = dihydrofolate reductase-thymidylate synthase,* PF3D7_0417200; *dhps = dihydropteroate synthetase,* PF3D7_0810800; *kelch13* = PF3D7_1343700. IPTp = Intermittent preventive therapy in pregnancy. *The *kelch13* mutations that may be associated with artemisinin partial resistance were taken from the MalariaGEN technical documents, accessed 2022-08-07, and are listed below:

- P441L, F446I, G449A, D452E, N458Y, C469Y, C469F, M476I, K479I, A481V, Y493H, R515K, S522C, P527L, N537I, N537D, G538V, R539T, I543T, P553L, R561H, V568G, P574L, R575K, M579I, C580Y, D584V, P667T, F673I, A675V, H719N
