# Supplementary material for: Drug resistance and vaccine target surveillance of Plasmodium falciparum using nanopore sequencing in Ghana
Source: Nat Microbiol. 2023 Nov 23;8(12):2365–77. doi: 10.1038/s41564-023-01516-6 (PMC10686832; doi:10.1038/s41564-023-01516-6)
Supplement: Supplementary file 2 — Reporting Summary [file 41564_2023_1516_MOESM2_ESM.pdf]

nature portfolio

Corresponding author(s): William L. Hamilton

Last updated by author(s): 15/09/23

Reporting Summary

Nature Portfolio wishes to improve the reproducibility of the work that we publish. This form provides structure for consistency and transparency in reporting. For further information on Nature Portfolio policies, see our Editorial Policies and the Editorial Policy Checklist.

Statistics

For all statistical analyses, confirm that the following items are present in the figure legend, table legend, main text, or Methods section.

n/a	Confirmed	
☐	☒	The exact sample size (*n*) for each experimental group/condition, given as a discrete number and unit of measurement
☐	☒	A statement on whether measurements were taken from distinct samples or whether the same sample was measured repeatedly
☐	☒	The statistical test(s) used AND whether they are one- or two-sided<br>*Only common tests should be described solely by name; describe more complex techniques in the Methods section.*
☒	☐	A description of all covariates tested
☐	☒	A description of any assumptions or corrections, such as tests of normality and adjustment for multiple comparisons
☐	☒	A full description of the statistical parameters including central tendency (e.g. means) or other basic estimates (e.g. regression coefficient) AND variation (e.g. standard deviation) or associated estimates of uncertainty (e.g. confidence intervals)
☐	☒	For null hypothesis testing, the test statistic (e.g. *F*, *t*, *r*) with confidence intervals, effect sizes, degrees of freedom and *P* value noted<br>*Give P values as exact values whenever suitable.*
☒	☐	For Bayesian analysis, information on the choice of priors and Markov chain Monte Carlo settings
☒	☐	For hierarchical and complex designs, identification of the appropriate level for tests and full reporting of outcomes
☒	☐	Estimates of effect sizes (e.g. Cohen's *d*, Pearson's *r*), indicating how they were calculated

Our web collection on statistics for biologists contains articles on many of the points above.

Software and code

Policy information about availability of computer code

Data collection	Nanopore sequencing and real-time base calling was performed using MinKNOW. For the venous blood samples, we used MinKNOW version 22.05.5, Bream 7.1.3, Configuration 5.1.5, Guppy 6.1.5, MinKNOW Core 5.1.0. For the dried blood spot samples, we used MinKNOW version 22.10.10, Bream 7.3.5, Configuration 5.3.8, Guppy 6.3.9, MinKNOW Core 5.3.1.
Data analysis	Nanopore data processing from fastq files to variant call files (VCF) was performed using the nano-rave pipeline, available at https://github.com/sanger-pathogens/nano-rave, including all dependent software packages. The nano-rave pipeline currently uses the following software (versions in brackets): bedtools (2.29.2), clair3 (1.0.0), freebayes (1.3.5), medaka (1.4.4), minimap2 (2.17), nanoplot (1.38.0), pycoqc (2.5.2), samtools (1.15.1), and tabix (1.11).<br><br>Downstream analyses were performed in R (version 4.2.1, 2022-06-23), using the tidyverse (1.3.1) and vcfR (1.13.0) packages; and in Python (version 3.8), using numpy (1.19.1), scipy (1.5.2), pandas (1.1.3), scikit-allel (1.3.5), matplotlib (3.4.2), and seaborn (0.11.0) packages. IGV version 2.13.10 was used to visualise sequence data.

For manuscripts utilizing custom algorithms or software that are central to the research but not yet described in published literature, software must be made available to editors and reviewers. We strongly encourage code deposition in a community repository (e.g. GitHub). See the Nature Portfolio guidelines for submitting code & software for further information.

Data

Policy information about availability of data

All manuscripts must include a data availability statement. This statement should provide the following information, where applicable:

- Accession codes, unique identifiers, or web links for publicly available datasets
- A description of any restrictions on data availability
- For clinical datasets or third party data, please ensure that the statement adheres to our policy

P. falciparum nanopore amplicon sequence data, with human genetic data removed, can be accessed from the ENA via study accession ERP145278, with sample metadata available in Supplementary Table 6. The P. falciparum reference sequences used were from the 3D7 v3.0 reference genome, accessed from PlasmoDB.

Research involving human participants, their data, or biological material

Policy information about studies with human participants or human data. See also policy information about sex, gender (identity/presentation), and sexual orientation and race, ethnicity and racism.

Reporting on sex and gender	Study refers to 'male', 'female', and 'unrecorded' participants - data collected at time of enrollment into the study.
Reporting on race, ethnicity, or other socially relevant groupings	Not applicable.
Population characteristics	For the prospectively collected venous blood samples (from Navrongo and Accra), there were 54 females, 51 males, and 4 unrecorded individuals. Median age was 12 years old (interquartile range 5-22 years). Population demographic data are not available for the retrospective set of dried blood spot (DBS) samples, collected from Navrongo in 2018. All patients had mild malaria (positive rapid diagnostic test with compatible clinical symptoms).
Recruitment	Patients presenting to clinics in and around Navrongo in northern Ghana, or to Ledzokuku Krowor Municipal Assembly (LEKMA) Hospital in Accra, and diagnosed with malaria with consistent symptoms and positive rapid diagnostic test (RDT) were eligible for recruitment into the study. Sampling bias may have been introduced by selecting patients with symptomatic malaria who presented for medical attention, as these samples may not be representative of the underlying parasite populations, including the large asymptomatic reservoir. Nonetheless, sampling of symptomatic malaria cases is technically more feasible than asymptomatic infections and can provide useful information such as drug resistance marker prevalence among symptomatic malaria cases.<br><br>Comparing between Accra and Navrongo may be confounded, as the samples from LEKMA Hospital may represent a more selected patient group with increased hospitalisation than those in the Navrongo community clinics. In addition, malaria transmission in Accra is lower than in Navrongo, where all the dried blood spot (DBS) samples were collected. Hence, intra-host genetic diversity and potential for outcrossing within the mosquito stage of the parasite life cycle may differ between these two sites, with more mixed infections likely to occur in Navrongo. However, we do not make strong claims on the comparison of circulating drug resistance alleles in these two sites.
Ethics oversight	The Navrongo samples were collected as part of the PAMGEN study, ethics approval ID: NHRCIRB343, obtained from the Navrongo Health Research Centre (NHRC) Institutional Review Board. This includes both the prospectively sequenced leucodepleted venous blood samples (collected in 2022) and the dried blood spot samples (collected in 2018). The LEKMA Hospital samples (collected in 2022) were collected as part of the EGSAT study, ethics ID: ECBAS030/21-22, approved by the College of Basic and Applied Sciences Ethics Review Committee, University of Ghana. All participants or their guardians (as appropriate) were provided detailed information sheets and gave informed consent prior to enrolment. Further approval was granted by the Wellcome Sanger Institute's Research Ethics Committee for the analysis of the samples. All patient-identifiable data are securely stored by Dr Amenga-Etego and only non-patient identifiable data were provided to the Wellcome Sanger Institute. The study complies with all relevant ethical regulations.

Note that full information on the approval of the study protocol must also be provided in the manuscript.

Field-specific reporting

Please select the one below that is the best fit for your research. If you are not sure, read the appropriate sections before making your selection.

☒ Life sciences ☐ Behavioural & social sciences ☐ Ecological, evolutionary & environmental sciences

For a reference copy of the document with all sections, see nature.com/documents/nr-reporting-summary-flat.pdf

Life sciences study design

All studies must disclose on these points even when the disclosure is negative.

Sample size	196 samples were nanopore amplicon sequenced, including 109 venous blood samples and 87 dried blood spots. No a priori sample size calculation was undertaken. However, the drug resistance marker and CSP allele frequencies calculated from the ONT data were compared

	against larger available datasets, such as the MalariaGEN Pf7 data resource (with >1,000 Ghanaian P. falciparum samples), and we confirmed the findings from ONT were consistent with these lager datasets.
Data exclusions	We applied selection criteria to which samples were used for nanopore sequencing. Venous blood samples from 33 patients were excluded from nanopore sequencing, due to low parasitaemia (<20 parasites per 200 white blood cells, WBC), poor DNA yield post-extraction (<1ng/µl) or due to time constraints. The 87 dried blood spot (DBS) samples were selected from a larger collection that had already passed MalariaGEN quality control (QC) filtering for Illumina whole genome sequencing, with the added requirements for parasitaemia to be microscopy positive i.e. >1 parasite per 200 WBC by thick film microscopy, and DNA concentration post-extraction to be >1ng/ul. Therefore, these samples were not representative of unselected symptomatic malaria cases in Ghana. These criteria were applied to reduce failure risk, maximising the cost-effectiveness of sequencing. However, we note that a parasitaemia cut-off of >1 parasite/ 200 WBC encompasses around 90% of symptomatic mild malaria cases in northern Ghana (based on data collected previously by Dr Amenga-Etego). Future work can assess nanopore performance on unselected, asymptomatic and/or ultra-low parasitaemia cases, for which failure rates would likely be higher.
Replication	15 samples were nanopore sequenced twice to confirm reproducibility of findings on repeat runs (14 venous blood samples and 1 dried blood spot). No discrepancies in the key drug resistance marker sites were identified using majority genotype calls.
Randomization	Randomization is not applicable to this study as it was not an intervention trial.
Blinding	Blinding is not applicable to this study.

Reporting for specific materials, systems and methods

We require information from authors about some types of materials, experimental systems and methods used in many studies. Here, indicate whether each material, system or method listed is relevant to your study. If you are not sure if a list item applies to your research, read the appropriate section before selecting a response.

Materials & experimental systems

n/a	Involved in the study
☒	☐ Antibodies
☒	☐ Eukaryotic cell lines
☒	☐ Palaeontology and archaeology
☒	☐ Animals and other organisms
☒	☐ Clinical data
☒	☐ Dual use research of concern
☒	☐ Plants

Methods

n/a	Involved in the study
☒	☐ ChIP-seq
☒	☐ Flow cytometry
☒	☐ MRI-based neuroimaging
